# SPUTNIK: an R package for filtering of spatially related peaks in mass spectrometry imaging data

**DOI:** 10.1093/bioinformatics/bty622

**Published:** 2018-07-13

**Authors:** Paolo Inglese, Gonçalo Correia, Zoltan Takats, Jeremy K Nicholson, Robert C Glen

**Affiliations:** Computational and System Medicine, Department of Surgery and Cancer, Imperial College London, London, UK

## Abstract

**Summary:**

*SPUTNIK* is an R package consisting of a series of tools to filter mass spectrometry imaging peaks characterized by a noisy or unlikely spatial distribution. SPUTNIK can produce mass spectrometry imaging datasets characterized by a smaller but more informative set of peaks, reduce the complexity of subsequent multi-variate analysis and increase the interpretability of the statistical results.

**Availability and implementation:**

*SPUTNIK* is freely available online from CRAN repository and at https://github.com/paoloinglese/SPUTNIK. The package is distributed under the GNU General Public License version 3 and is accompanied by example files and data.

**Supplementary information:**

Supplementary data are available at *Bioinformatics* online.

## 1 Introduction

Over the last few years, mass spectrometry imaging (MSI or IMS) has demonstrated great potential in discovering and elucidating chemical processes in a wide variety of research contexts. MSI has been used to determine possible cancer biomarkers (Franck *et al.*, 2009), and recent technologies are capable of detecting molecular signals at the cellular level (Kompauer *et al.*, 2017). The properties of such data type, such as high ion dimensionality and the presence of noise fluctuations in the spectral profiles, make the pre-processing phase and the extraction of informative features for the subsequent statistical analysis extremely important.

Software packages, such as *MALDIquant* (Gibb and Strimmer, 2012) can filter peaks based on the presence of signals in a minimum fraction of samples, but unfortunately these filters do not to take into account the information contained in the spatial localization of the signals as addressed in recent work (Alexandrov and Bartels, 2013; Fonville *et al.*, 2012; Palmer *et al.*, 2017).

*SPUTNIK (SPatially aUTomatic deNoising for Ims toolKit)* provides a series of filters which aim select meaningful and informative peaks, based on the plausibility of their spatial distributions, given the information about the signal source (Supplementary Material S1, Table S1). It provides an estimation of split peaks, a correlation-based filter, a pixel count based filter and a series of tests based on complete spatial randomness. Each class of filters is designed to remove uninformative peaks based on specific assumptions. An example of the effects of each filter (with the default parameters) on the final dimensionality of two example datasets (MALDI-MSI and DESI-MSI) is shown in Supplementary Material S1, Figure S1. *SPUTNIK* is freely distributed as an R package written using S4 object-oriented programming.

Two example workflows showing how to apply SPUTNIK to both MALDI-MSI and DESI-MSI datasets are provided together with the package (Supplementary Materials S2 and S3). The original imzML and the associated optical image files for the MALDI-MSI dataset are available at https://www.ebi.ac.uk/pride/archive/projects/PXD001283/.

**Fig. 1.**
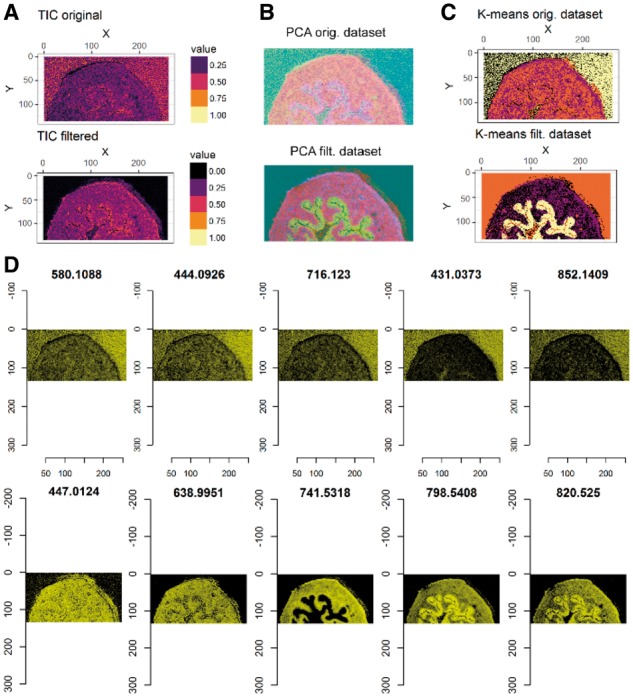
A comparison between the original and the filtered MALDI-MSI dataset (Rompp *et al.*, 2010): (A) total ion count (TIC) images, (B) RGB images of the three first principal components scores scaled in [0, 1] and (C) results of k-means clustering with four clusters applied to the PCA scores responsible for 95% of the total variance. The heatmaps (D) show the intensities of five filtered (top row) and five selected peaks (bottom row) with the five largest average intensities (Supplementary Material S1, Fig. S6). The images of the filtered peaks show that they are mainly localized outside of the tissue region. All the results confirm that the filters reduce the effect of signal noise and allow a clear identification of tissue sub-structures

An example of the application of the SPUTNIK pipeline to MALDI-MSI from mouse urinary bladder specimen (Rompp *et al.*, 2010) is shown in Figure 1 (see complete workflow in Supplementary Material S2). The sum of the matched peaks intensities (pre-processed using MALDIquant) was used as a reference for determining the tissue specimen location and ROI detection. The ROI quality was visually evaluated comparing its morphology with an external tissue image (e.g. optical image of H&E stained tissue). The dataset was filtered using a correlation-based filter (reference = binary ROI calculated by k-means, measure = Spearman’s correlation, threshold = 0); followed by a count pixels filter with a minimum number of connected pixels equal to 4. Finally, a complete spatial randomness filter was applied using the Kolmogorov-Smirnov test with the total ion count image as a covariate; Bonferroni corrected *P*-values (α = 0.001) were used to select the peaks. The pipeline was capable of reducing the dimensionality from 1175 peaks to 204 peaks. Visualization of the filtered dataset and analyses based on the reduced number of peaks result in images with enhanced contrast (Fig. 1B and C). This also provided improved clustering results, with an enhanced contrast due to the removal of signals associated with noise. A complete workflow of the application of SPUTNIK on the example DESI-MSI dataset is available in Supplementary Material S3.

## 2 Algorithms details

### 2.1 Split peak estimation

Random peak shifting can generate false multiple peaks during the peak-matching procedure. These peaks signals, which represent the same ion source, are assigned to multiple m/z values. In order to identify the occurrence of this issue, we hypothesized that split peaks are randomly assigned to contiguous m/z values within the limits of instrumental error. Additional conditions to assign multiple peaks to the same m/z value are: (i) their peak intensity signals are localized in small or non-overlapping spatial regions, (ii) at least one of the peaks signal images shows a sufficient level of ‘spatial regularity’, (iii) the combined signal, generated by merging the intensities of the candidate split peaks, is associated with an image with a spatial regularity at least as high as the images associated with the original peaks. Spatial regularity measures available are: (i) ratio of scattered pixels (defined as the number of Otsu’s thresholded (Otsu, 1975) disconnected signal pixels divided by the total number of signal pixels), (ii) spatial chaos (Palmer *et al.*, 2017), (iii) Gini index (Hurley and Rickard, 2009). An example of simulated split peak merging from the DESI-MSI sample is shown in Supplementary Material S1, Figure S2. Based on its purpose, the split peak tool should always be applied before any other filtering tool.

### 2.2 Reference similarity filter

Often, signals derived from non-informative peaks (e.g. matrix or solvent related peaks) are characterized by an unrelated spatial distribution compared to the geometrical shape of the expected signal source (e.g. a tissue section). In order to identify and remove these peaks, we designed a filter based on the similarity between the peak intensity images and a reference image. Available measures to estimate the similarity between the peak and the reference signal distributions are: Pearson’s correlation, Spearman’s correlation, structural similarity index measure (Wang *et al.*, 2004) and normalized mutual information. Two options are available for calculating the reference signal, a continuous measure among ‘sum’, ‘median’, ‘mean’ or ‘first principal component scores’ of the entire set of peak intensities, and a binary mask, representing the region of interest (ROI), calculated either applying Otsu’s thresholding to the reference signal seen as an image, or applying k-means clustering with two clusters on the entire dataset. Additionally, external reference and ROI images can be used, after opportunely resizing and registering them with the MS image. The command ‘msImage’ allows to easily convert arbitrary images represented as pixel intensity matrices into MS images compatible with SPUTNIK (an example of the filter applied using the ROI generated by the H&E optical image registered with the sum of the ion intensities in the 800–900 m/z range is shown in Supplementary Material S1, Fig. S3). By default, a similarity threshold equal to 0 guarantees that ions also localized in small regionswithin the ROI are not filtered. Scaled first three principal components scores image of the filtered peaks data confirm that the filter successfully removes the ions localized outside of the tissue in the DESI-MSI and MALDI-MSI examples (Supplementary Material S1, Fig. S4). When off-tissue regions are available, the user should run the reference similarity filter before all the other filters. In this way, the dataset dimensionality can be significantly reduced, increasing the global contrast between tissue and off-tissue regions.

### 2.3 Pixel count based filter

The Poisson spatially distributed signals (due to shot-like noise) are characterized by a more scattered spatial distribution than the real signal. Meaningful clusters of pixels should be larger than the expected smallest spatial sub-regions. Under this assumption, we designed a filter that takes into account the number of connected pixels where the peak intensity is higher than the background level. Using a binary ROI mask, calculated similarly to that described in the ‘Reference similarity filter’ section or generated externally (e.g. binarization of the registered optical image of the H&E stained tissue), the connected signal sub-regions are detected from Otsu’s thresholded binary peak image within the ROI. Subsequently, the filter selects those peaks characterized by connected regions larger than the provided number of pixels, which are user-defined based on the expected smallest meaningful image sub-regions. Different levels of ‘aggressiveness’ take into account the clusters size which is also outside of the ROI. When off-tissue regions are not present, pixel count based filter can be used with an ROI consisting of a matrix of all ones (Supplementary Material S1, Fig. S5).

### 2.4 Complete spatial randomness filter

A further filter is based on the rejection of the null hypothesis of a peak signal following a complete spatial random distribution. The assumption behind these statistical tests is that a non-informative peak signal is spatially distributed as a homogeneous spatial Poisson process (Maimon and Rokach, 2010). The tests use Otsu’s thresholded binary pixels associated with the peak signal to define a two-dimensional point pattern process; two tests are currently available: (i) *Clark Evans test* (Clark and Evans, 1954), (ii) *Kolmogorov-Smirnov* test against a covariate distribution (Berman, 1986), calculated with the same methods used to extract the reference image. The tests are based on the already available *spatstat* R package (Baddeley and Turner, 2005). This represents the least aggressive filter, since it does not take into account of the connectivity between pixels and the tissue spatial distribution, but only tests whether ion intensities reflect the overall contrast between the tissue and off-tissue regions. Similarly to the count pixel filter, this filter should be used when off-tissue regions are not available (Supplementary Material S1, Fig. S5).

More details about the algorithms are available in Supplementary Material S1.

## 3 Conclusions

*SPUTNIK* provides a collection of flexible filters for the detection of peaks associated with non-realistic spatial distributions, given the prior information about the signal source localization.

Two tutorials, distributed with the package, show how to apply the filtering pipeline to MALDI-MSI and DESI-MSI datasets.

## Supplementary Material

Supplementary Data 1

Supplementary Data 2

Supplementary Data 3
