# Supplementary material for: SPUTNIK: an R package for filtering of spatially related peaks in mass spectrometry imaging data
Source: Bioinformatics. 2018 Jul 13;35(1):178–80. doi: 10.1093/bioinformatics/bty622 (PMC6298046; doi:10.1093/bioinformatics/bty622)
Supplement: Supplementary Data 1 [file bty622_supplementary_data_1.pdf]

SPUTNIK: an R package for filtering of spatially related peaks in mass spectrometry imaging data.

Paolo Inglese[†], Gonçalo Correia[†], Zoltan Takats[†], Jeremy K Nicholson[†], Robert C Glen[†]

[†] *Computational and Systems Medicine, Department of Surgery and Cancer Imperial College London, London, UK*

Supplementary Data 1

Split peaks estimation algorithm

Split peaks estimation algorithm searches for candidate split peaks in the provided vector of the m/z values associated with the matched spectral peaks of the dataset.

Arguments:

1. "*mzTolerance*": *m/z measurement precision tolerance.* Maximum distance in ppm to consider two peaks contiguous. This should be lower or equal to the expected instrumental error;
2. "*sharedPixelRatio*": *shared signal pixels ratio*. Ratio between the number of signal (non-background) pixels shared by the selected contiguous peaks. The signal pixels are obtained from the binarized peak intensity image using Otsu's thresholding (Otsu, 1975);
1. "*sparseness*": *image regularity measure*. Available measures are: Gini index (Hurley and Rickard, 2009), scatter ratio (defined as the ratio of the number of signal pixels and the total number image pixels), spatial chaos (Palmer, et al., 2017);
2. "*threshold*": *sparseness cut-off value.*

Algorithm:

1. scan for groups of peaks whose m/z values are within *mzTolerance*;
2. for each group of peaks:
    a. for each peak, determine which pixels contain signal using Otsu's thresholding;
    b. calculate the ratio *R* of the common signal pixels for the selected peaks;
    c. if *R* < *sharedPixelRatio*:
        i. calculate the image's regularity *S* using the *sparseness* measure;
        ii. if at least one image has *S* < *threshold*:
            1. create a new image whose pixels' intensity is the sum of the intensities of peaks in the group;
            2. if *sparseness* of the combined image is less or equal to the maximum *sparseness* of the group peaks images:
                a. replace the group with the combined ion feature. The average m/z value is assigned to the new feature.

Reference image calculation

SPUTNIK provides two types of reference images that can be used for identifying the region of interest: a continuous and a binary valued image.

The following methods are provided to calculate the *continuous* valued reference image:

1. "*sum*": the intensity value for each pixel is calculated as the sum of individual peaks intensities (total ion count) of the corresponding spectrum;
2. "*mean*": the intensity value for each pixel is calculated as the average of the non-zeros intensities of the corresponding peaks spectrum;
3. "*median*": analogous to *mean*, using *median* values;
4. "*pca*": the set of spectra from the entire dataset is used to train a principal component analysis (PCA) model. The reference image pixels intensities correspond to the scores of the first principal component.

The binary valued reference image can be calculated using two methods:

3. "*otsu*": Otsu's method is used to automatically determine the threshold to binarize the reference image calculated using one of the previous methods for continuous valued reference generation;
4. "*kmeans*": k-means clustering with 2 clusters is applied on the entire dataset. The two clusters labels assigned to each pixel are used as the image binary intensities.

Reference similarity filter

Reference similarity filter selects peaks whose intensities are distributed similarly to a reference signal, generally representing the region of interest (eg. a tissue section).

Arguments:

1. *"referenceImage": reference image calculated using the methods in "Reference image calculation";*
2. *"method": similarity measure.* Available methods are: Pearson's correlation, Spearman's correlation, structural similarity index measure (SSIM) (Wang, et al., 2004), and normalized mutual information (NMI).
3. *"threshold": similarity cut-off value (default = 0).*

Algorithm:

1. calculate the reference image using one of the methods decribed in "Reference image calculation";
2. for each peak intensity:
    a. calculate the similarity measure between the peak intensity and the reference image;
3. select peaks with a similarity measure greater than *threshold.*

Pixel count based filter

Pixel count based filter select peaks whose signal pixels are connected forming groups larger than a given threshold. The threshold value is related with the physical size of the expected smallest sub-region of interest.

Arguments:

1. *"roiImage": binary image representing the region of interest*. This can be calculated using one of the methods described in "Reference image calculation";
2. *"minNumPixels": the minimum number of connected pixels to select the peak;*
3. *"aggressive": level of "aggressiveness".*

Algorithm:

4. for each peak binarized image (using Otsu's thresholding):
    a. if *aggressive = 0:*
        i. measure the largest size *N* of connected regions (number of pixels in each cluster);
        ii. if *N* is larger than *minNumPixels:*
            1. retain the peak;
    b. if *aggressive = 1:*
        i. measure the largest size *N1* of connected regions within the ROI and the largest size *N2* of connected regions outside the ROI, as defined by *roiImage;*
        ii. if *N1* is greater than *minNumPixels AND N1* is greater than or equal than *N2*:
            1. retain the peak;
    c. if *aggressive = 2:*
        i. measure the largest size *N1* of connected regions within the ROI and the largest size *N2* of connected regions outside the ROI, as defined by *roiImage;*
        ii. if *N1* is greater than *minNumPixels AND N1* is smaller than *minNumPixels*:
            1. retain the peak.

Complete spatial randomness filter

Complete spatial randomness filter selects peaks whose signals distributions reject the null hypothesis of complete spatial randomness.

Arguments:

1. *"method"*: *statistical test.* Available methods are: Kolmogorov-Smirnov *"KS"* test (Baddeley and Turner, 2005) and Clark Evans *"ClarkEvans"* test (Clark and Evans, 1954).

Algorithm:

1. If *method = “KS”:*

    a. use the reference image, calculated using the methods described in “Reference image calculation”, as covariate density;

    b. for each peak:

        i. define a point pattern process (Baddeley and Turner, 2005) from the Otsu’s binarized peak image;

        ii. calculate the p-value

2. if *method = “ClarkEvans”*:

    a. for each peak:

        i. define a point pattern process (Baddeley and Turner, 2005) from the Otsu’s binarized peak image;

        ii. apply Clark Evans test to calculate the p-value.

3. Correct the p-values using multiple testing correction method.

4. Peaks are selected setting a threshold for the p-values.

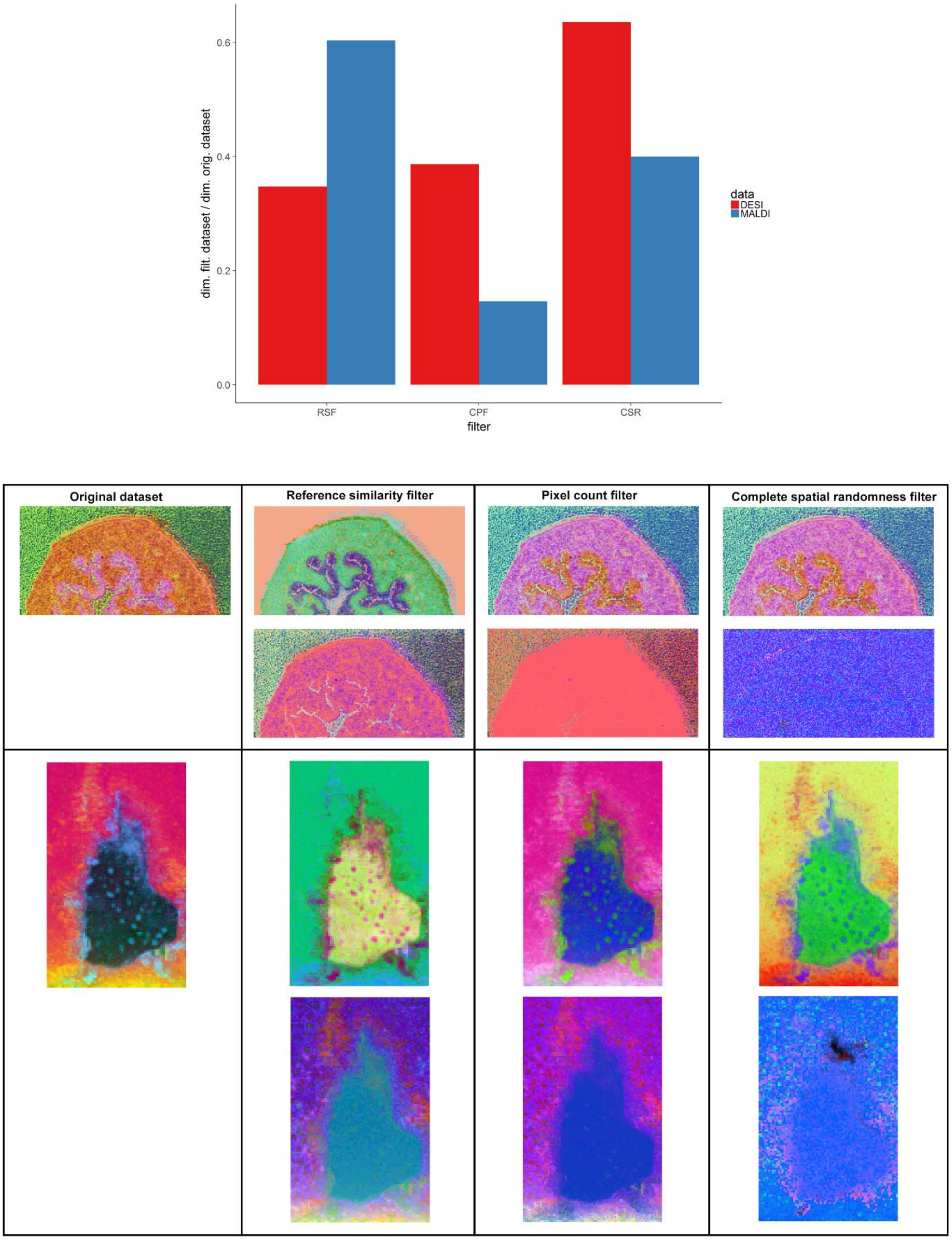

Figure S1 – Effect of single filter applied to the two example dataset provided with the package. The filters were applied with the default parameters. The results confirm that DESI-MSI peak images are less scattered than MALDI-MSI, since the reference similairy based filter (RSF) returns a smaller dataset than the pixel count based filter (CPF) and the complete spatial randomness (CSR) filter. On the contrary, MALDI-MSI suffers more of highly scattered peak images, as shown by the relatively smaller

dimensionality of the dataset after applying CPF. This difference can be due to a higher spatial resolution or a greater effect of the applied matrix in MALDI-MSI. (Code is available in the 'Code section' of the Supplementary Data 1). In the bottom table: effect of the single filters on the first 3 principal components of the MALDI-MSI and DESI-MSI datasets. PCA scores are displayed as [0, 1] scaled RGB channels. PCA of the original datasets (first column) are compared with those after applying the 3 filters independently using the k-means ROI and the default parameters (columns 2, 3, 4). For each filter, both the PCA of the retained peaks (top) and filtered peaks (bottom) are shown, confirming that the filters remove signals unrelated with the tissue.

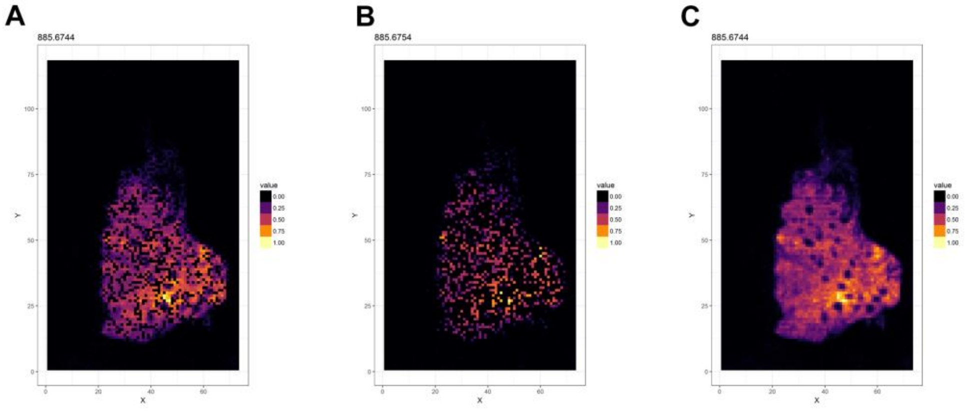

Figure S2 – Example of split peak merging. The peak at 885.6744 m/z (A) was artificially split into an additional peak at 885.6754 m/z (B), associated with the intensity of randomly selected 30% of the original image pixels. After applying the filter, the original image is reconstructed (C).

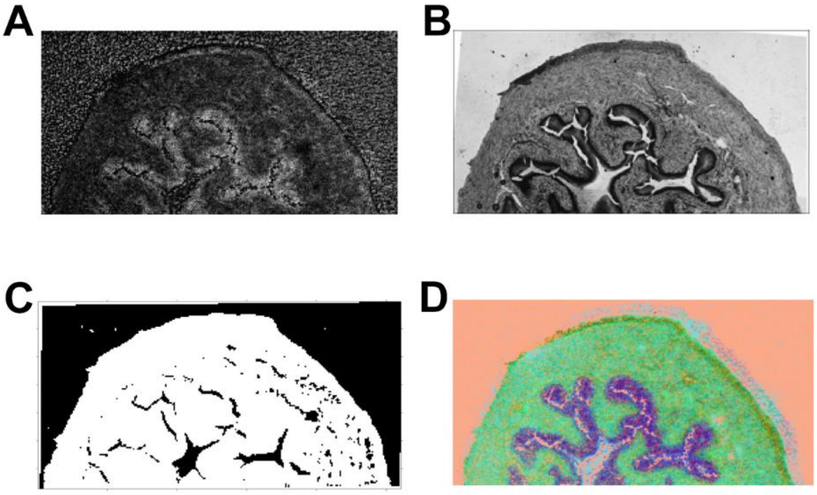

Figure S3 – The reference similarity filter can be also applied using an external reference image. Here, the ROI is generated binarizing the H&E gray scaled optical image (B), after registering it with the sum of the ion intensities in the 800-900 m/z range (A). The registration was performed applying an affine transformation on the optical image using the ions image as template. The binary mask of the registered H&E image (C) was used as ROI. The RGB image of the [0, 1] scaled first 3 principal components (D) shows that, after removing 664 of the 1175 peaks, the overall signal is more informative about the tissue.

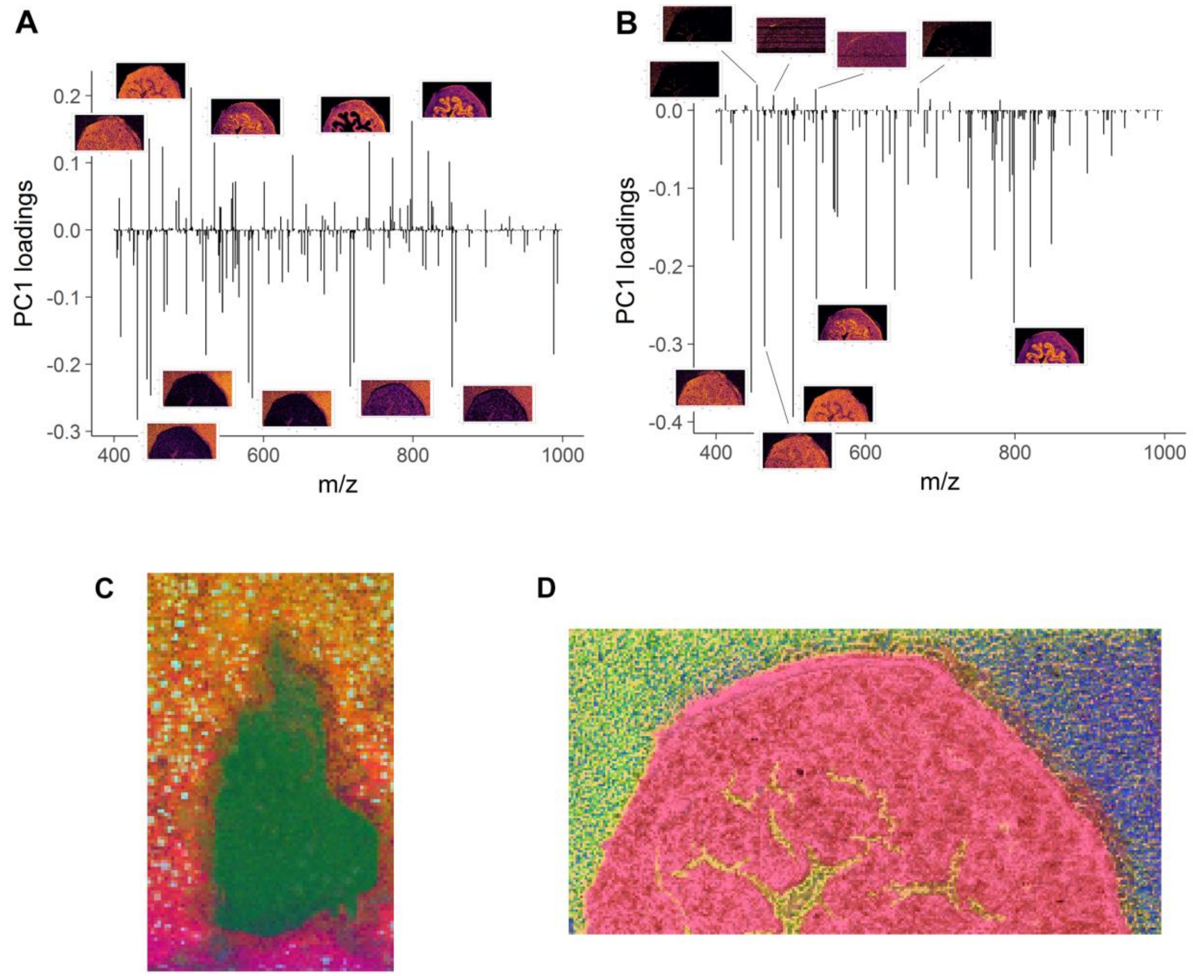

Figure S4 – Effect of the reference similarity filter on the first principal component loadings shows that the filter effectively removes the ions associated with the off-tissue region for the MALDI-MSI dataset (negative loadings in the original dataset (A)). Indeed, the loadings calculated from the filtered dataset (B) show that most of the variance explained by the first principal component is associated with the tissue region (negative loadings), and the off-tissue ions are associated with very small loading values. The small images represent the scaled intensity images of the 5 peaks with the highest and lowest loading values. These results are confirmed by the RGB image corresponding to the [0, 1] scaled first 3 principal components of the filtered ions on the DESI-MSI (C) and MALDI-MSI (D) datasets. The spatial distributions show that the filter removed ions that were mainly localized outside of the tissue section with no loss of significant sub-structures associated with the tissue.

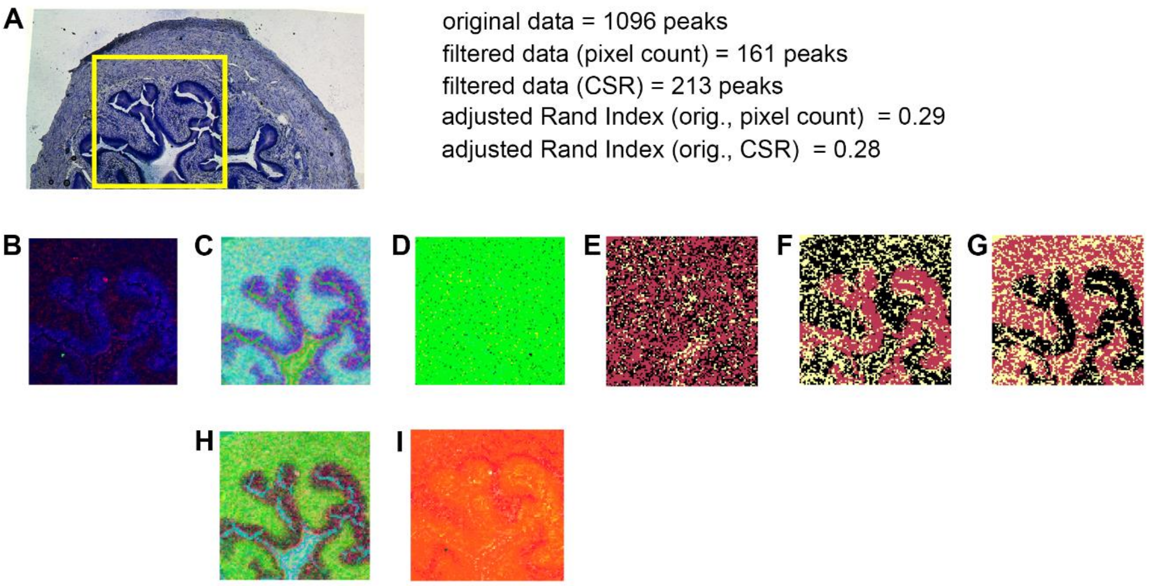

Figure S5 – Example of individual filters applied to a sub-region of the MALDI-MSI dataset without an off-tissue region. Here the only the pixels within the yellow rectangle (A) were analysed. Pixel count filter was applied with the following parameters: minimum size of connected sub-regions = 9, 'aggressiveness' equal to 0 was used. A matrix of all ones was used as ROI image using the 'msImage' command. The complete spatial randomness filter was applied using the 'ClarkEvans' test. The RGB image corresponding to the [0, 1] scaled first 3 principal components of the original data (B) shows less contrasted patterns than those after the pixel count filter (C) (final size = 161 peaks) and the CSR filter (H) (final size = 213 peaks). On the contrary, the RGB image corresponding to the removed peaks corresponds to an unstructured image for both the filters (D, I). K-means (3 clusters) of the scores of the principal components explaining the 95% of variance resulted in clear structures resembling the tissue morphology for the filtered data (F, G). Similar structures were not captured by k-means applied to the scores of the original data (E). The adjusted Rand index confirmed the difference between the clusters of the original and filtered data. This result suggests that both the filters were able to increase the quality of the unsupervised analysis, while significantly reducing the dimensionality of the data.

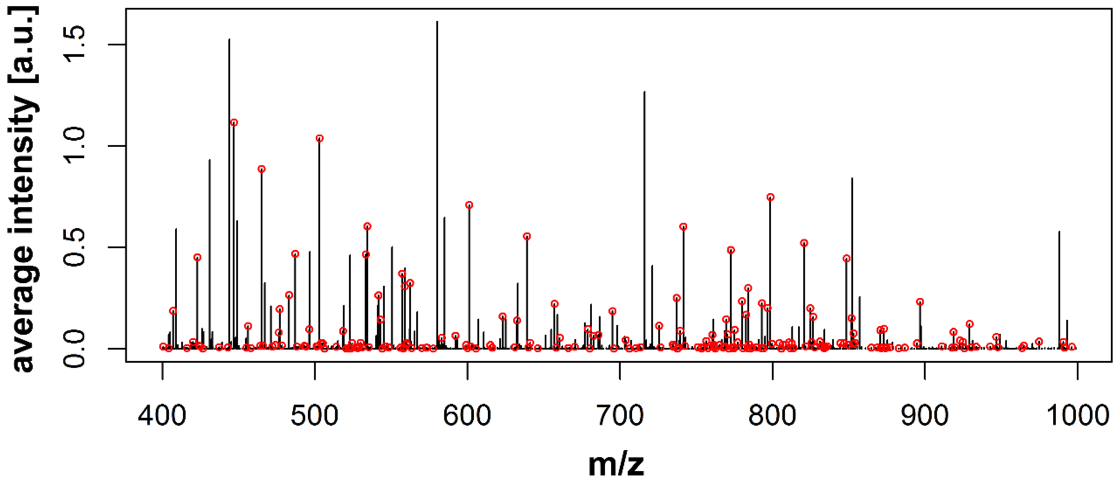

Figure S6 – Average mass spectrum of the mouse urinary bladder MALDI-MSI dataset. The red dots represent the 204 selected peaks. The shown intensities are calculated as the average of all the pixels spectra intensities.

**Filter family**	**Method**	**Rationale**
Reference similarity	Tests for ion spatial distributions similar to a given reference (heatmap or binary ROI).	Most MSI samples are constituted by a background region which contains solvent/matrix and contaminants signals, and a tissue region, which contains signals of biological nature. The provided set of filters exploit this assumption, removing all the ions whose spatial distribution is not confined in the provided reference mask, representing the spatial distribution of the tissue.
Pixel count	Tests for disconnected ion signal pixels patterns or connected pixel regions smaller than a user-defined threshold	Noise signals are characterized by a scattered random patterns. These patterns can be associated with detector noise, when the signal is not present in the source or when the source signal is low intense (close to the detection limits). These issues make these signals unreliable for statistical analysis. The smallest allowed connected region is data-dependent (spatial resolution, prior knowledge on the expected granularity of the spatial signal patterns). By default, this threshold is set equal to 9 pixels.
Complete spatial randomness	Tests for the randomness of the spatial distribution of the signal pixels. Given a reference, it tests whether	Complete spatial randomness allows the identification of (even disconnected) pixel patterns that are statistically non-random or that are characterized by a spatial density that reflects an external

	the (even disconnected) pixel patterns covary with it.	reference heatmap. This set of filters can be used when scattered patters can still represent sample related signals. In that case, these filters should be used instead of the pixel count based filters.

Table S1 – Scheme of the three main filter families provided with SPUTNIK. Each family measures different properties of the spatial distribution of the peaks (Method column), and it addresses specific characteristics expected in the noise/uninformative signals (Rationale column).

References

Baddeley, A. and Turner, R. spatstat: An R package for analyzing spatial point patterns. *Journal of Statistical Software* 2005;12(6):1-42.

Clark, P.J. and Evans, F.C. Distance to Nearest Neighbor as a Measure of Spatial Relationships in Populations. *Ecology* 1954;35(4):445-453.

Hurley, N. and Rickard, S. Comparing Measures of Sparsity. *Ieee Transactions on Information Theory* 2009;55(10):4723-4741.

Otsu, N. A threshold selection method from gray-level histograms. *Automatica* 1975;11(285-296):23-27.

3. Palmer, A.*, et al.* FDR-controlled metabolite annotation for high-resolution imaging mass spectrometry. *Nat Methods* 2017;14(1):57-60.

4. Wang, Z.*, et al.* Image Quality Assessment: From Error Visibility to Structural Similarity. *IEEE Transactions on Image Processing* 2004;13(4):600-612.

Code section

Script used to evaluate the effect of single filters on the example datasets.

```
library(SPUTNIK)

library(ggplot2)

## Load data ------------------------------------------------------------------

maldiData <- SPUTNIK::bladderMALDIRompp2010(verbose = T)
```

```
maldiData[is.na(maldiData)] <- 0

desiData <- SPUTNIK::ovarianDESIDoria2016(verbose = T)
desiData[is.na(desiData)] <- 0

shape <- attr(maldiData, 'size')
mz <- attr(maldiData, 'mass')
msi_maldi <- msiDataset(maldiData, mz, shape[1], shape[2])

shape <- attr(desiData, 'size')
mz <- attr(desiData, 'mass')
msi_desi <- msiDataset(desiData, mz, shape[1], shape[2])

rm(mz, shape)

## Normalize ----------
msi_maldi <- normIntensity(msi_maldi, 'median')
msi_maldi <- varTransform(msi_maldi, 'log')
msi_desi <- normIntensity(msi_desi, 'median')
msi_desi <- varTransform(msi_desi, 'log')

## Global peak filter
ref_roi_maldi <- refAndROIimages(msi_maldi, refMethod = 'sum', roiMethod = 'kmeans')
ref_roi_desi <- refAndROIimages(msi_desi, refMethod = 'sum', roiMethod = 'kmeans')

plot(ref_roi_maldi$ROI)
plot(ref_roi_desi$ROI)

## If necessary, invert the ROI
ref_roi_maldi$ROI <- invertImage(ref_roi_maldi$ROI)
ref_roi_desi$ROI <- invertImage(ref_roi_desi$ROI)

## Compare the effect of single filters on MALDI and DESI using the default parameters
gpf_maldi <- globalPeaksFilter(msi_maldi, referenceImage = ref_roi_maldi$ROI)
gpf_desi <- globalPeaksFilter(msi_desi, referenceImage = ref_roi_desi$ROI)

cpf_maldi <- countPixelsFilter(msi_maldi, roiImage = ref_roi_maldi$ROI)
cpf_desi <- countPixelsFilter(msi_desi, roiImage = ref_roi_desi$ROI)

csr_maldi <- CSRPeaksFilter(msi_maldi)
```

```
csr_desi <- CSRPeaksFilter(msi_desi)


df <- data.frame(x = factor(c(rep('gpf', 2),
                       rep('cpf', 2),
                       rep('csr', 2)), levels = c('gpf', 'cpf', 'csr')),
                 y = c(length(gpf_maldi$sel.peaks) / ncol(maldiData), length(gpf_desi$sel.peaks) /
ncol(desiData),
                       length(cpf_maldi$sel.peaks) / ncol(maldiData), length(cpf_desi$sel.peaks) /
ncol(desiData),
                       sum(csr_maldi$q.value < 0.001) / ncol(maldiData), sum(csr_desi$q.value < 0.001)
/ ncol(desiData)),
                 data = factor(c('MALDI', 'DESI', 'MALDI', 'DESI', 'MALDI', 'DESI')))


gg <- ggplot(df, aes(x = x, y = y, fill = data)) + geom_bar(stat = 'identity', position = 'dodge') +
  scale_x_discrete(labels = c('RSF', 'CPF', 'CSR')) + scale_fill_brewer(palette = 'Set1') +
  xlab('filter') + ylab('dim. filt. dataset / dim. orig. dataset') + theme_classic(base_size = 24)
plot(gg)
```
