# Supplementary material for: SPUTNIK: an R package for filtering of spatially related peaks in mass spectrometry imaging data
Source: Bioinformatics. 2018 Jul 13;35(1):178–80. doi: 10.1093/bioinformatics/bty622 (PMC6298046; doi:10.1093/bioinformatics/bty622)
Supplement: Supplementary Data 2 [file bty622_supplementary_data_2.pdf]

SPUTNIK MALDI-MSI workflow

Contents

Merge estimated split peaks . . . 2
Determine the reference image and the ROI . . . 3
Global similarity filter . . . 6
Connected pixels count based filter . . . 7
Complete spatial randomness filter . . . 7
Comparison between the original and filtered datasets . . . 10
Verify selected peaks . . . 16
References . . . 18

```r
library("SPUTNIK")
library("imager", quietly = T)
```

```
##
## Attaching package: 'imager'

## The following object is masked from 'package:magrittr':
##
##     add

## The following object is masked from 'package:plyr':
##
##     liply

## The following objects are masked from 'package:stats':
##
##     convolve, spectrum

## The following object is masked from 'package:graphics':
##
##     frame

## The following object is masked from 'package:base':
##
##     save.image
```

IMPORTANT! Do not normalize the peaks intensity matrix before running the `splitPeaksFilter`. Also the variance stabilizing transformation should be applied after the filter. The MALDI MSI data set (Römpp et al. 2010) acquired with Orbitrap MS in positive ion mode has been pre-processed using [MALDIquant](https://github.com/cran/MALDIquant) (Gibb and Strimmer 2012). No filter was applied during the pre-processing. The intensity matrix has been loaded with the variable name `maldiData`. The number of pixels is equal to 260 x 134 (x, y). MALDIquant assign a NA to empty peak signals, these should be converted into zeros before running the analysis.

```r
## Load the MALDI-MSI dataset
maldiData <- bladderMALDIRompp2010()
```

```
## Downloading the data from the repository...
## Loading the data in the R environment...
```

```r
maldiData[is.na(maldiData)] <- 0
```

```r
## Image size
sz <- attr(maldiData, "size")
```

```
## Print the original dimensions of intensity matrix
print(dim(maldiData))
```

```
## [1] 34840  1175
```

```
## Create the MSI dataset
msiX <- msiDataset(values = maldiData, mz = attr(maldiData, 'mass'),
                   rsize = 260, csize = 134)
```

Merge estimated split peaks

Firstly, we test whether some peaks have been split during the matching process. This can be due to issues with the spectra alignment. Usually, this error generates close peaks that are spatially localized in complementary regions. In the `splitPeaksFilter`, the smallest distance that defines close peaks is expressed in ppm. This value should be carefully chosen to be close to the instrumental error; a too large value will produce a wrong merging. The `sharedPixelsRatio` represents the maximum accepted ratio between the pixels shared by two or more peaks and the total number of peaks signal pixels.

Estimate the split peaks.

```
split.peaks <- splitPeaksFilter(msiData = msiX,
                                mzTolerance = 10,
                                sharedPixelsRatio = 0,
                                sparseness = "scatter.ratio",
                                threshold = 0.5,
                                returnDetails = T,
                                verbose = T)
```

```
## Determining close peaks. This may take a while...
## Min. distance between peaks (ppm) = 6.904487
## Found 6 groups of peaks to test for scatteredness.
## Testing scatteredness of merged images...
## No peak groups to merge found.
```

In this case, no groups of peaks have been merged, since no image to merge was structured.

Now, we can normalize the intensities with median scaling and apply the log-transformation. Let's apply the transformations also to the original matrix for a subsequent comparison of the results.

```
Start.time <- Sys.time()

maldiData[maldiData == 0] <- NA

for (i in 1:nrow(maldiData))
{
  maldiData[i, ] <- maldiData[i, ] /
    median(maldiData[i, ], na.rm = T)
}

maldiData[is.na(maldiData)] <- 0

maldiData <- log(maldiData + 1)

Sys.time() - Start.time
```

```
## Time difference of 5.121284 secs
```

Normalize the msiX matrix elements.

```
# Apply median scaling and log-transformation
start.time <- Sys.time()

msiX <- normIntensity(msiX, "median")
msiX <- varTransform(msiX, "log")

Sys.time() - start.time
```

```
## Time difference of 4.514943 secs
```

Determine the reference image and the ROI

Before, extracting the reference image and the ROI, we to select the measure to be used to calculate the reference intensities among `sum`, `mean`, `median`, and `pca`. Furthermore, we have to provide the method used to generate the ROI, among `otsu` and `kmeans`.

Sometimes, the region of interest may be characterized by a lower intense TIC. In these circumstances, the argument `invertRef` must be set to `TRUE`. Here, also, we apply the `kmeans` binarization.

```
ref.roi <- refAndROIimages(msiData = msiX,
                           refMethod = "sum",
                           roiMethod = "kmeans", # <== BINARIZE
                           useFullMZRef = TRUE,
                           smoothRef = TRUE,
                           invertRef = TRUE, # <== INVERT COLORS
                           verbose = TRUE)
```

```
## Plot reference (continuous) image
plot(ref.roi$Reference)
```

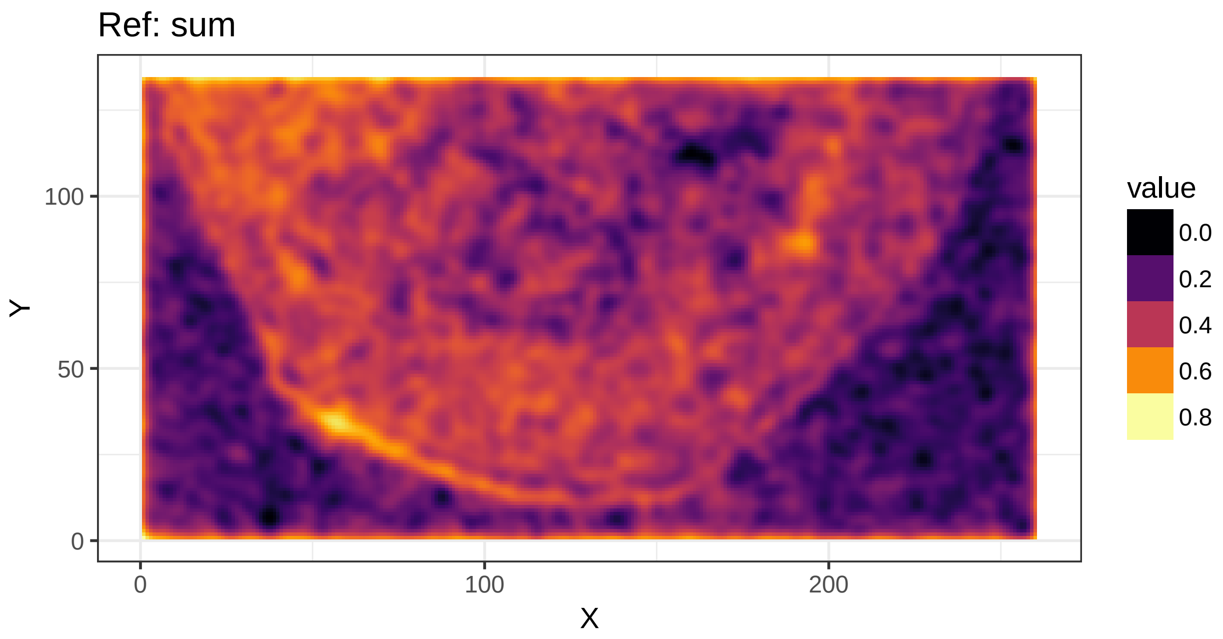


```
## Plot ROI mask (binary) image
plot(ref.roi$ROI)
```

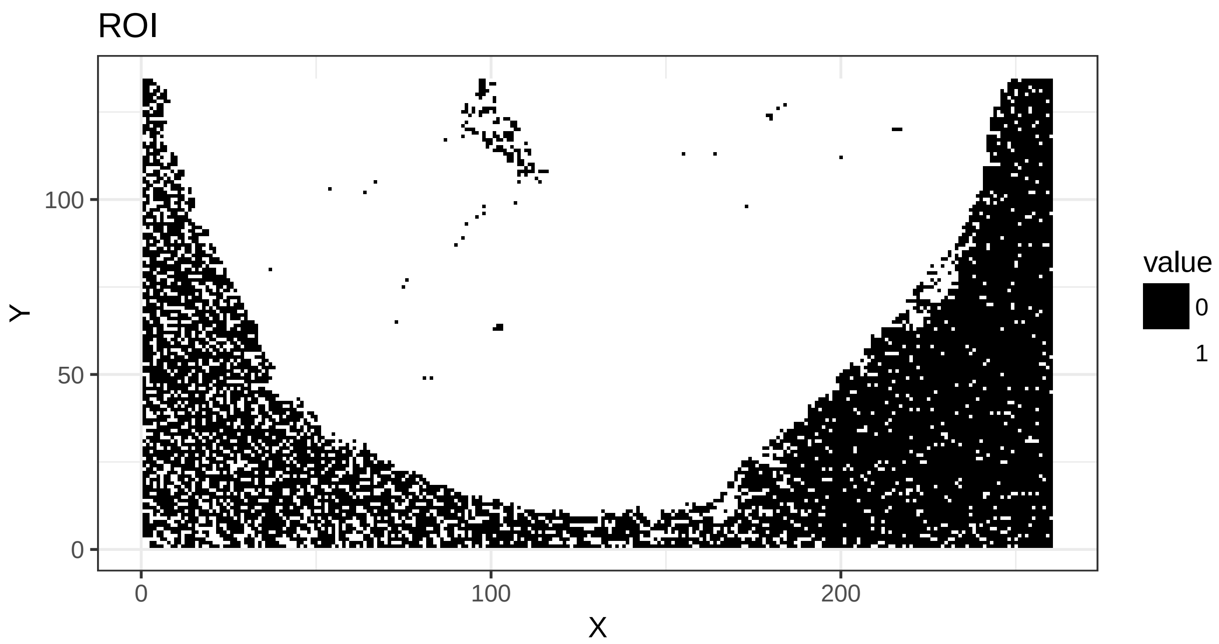

K-means cluster labels are assigned randomly. Let's be sure that the value 1 is assigned to the ROI. A heuristic approach is to check whether the majority of pixels randomly selected from the expected ROI are assigned to 1.

```
## Rect boundaries
x.int <- c(50, 100)
y.int <- c(100, 150)
num.pixels <- 200
rand.x <- sample(c(x.int[1]:x.int[2]), num.pixels, replace = T)
rand.y <- sample(c(y.int[1]:y.int[2]), num.pixels, replace = T)
rand.value <- ref.roi$ROI@values[cbind(rand.y, rand.x)]

table(rand.value)
```

```
## rand.value
##   1
## 200
```

```
## If the majority of pixels has been assigned to 0, invert the ROI
if (names(table(rand.value))[which.max(table(rand.value))] != "1")
{
  ref.roi$ROI <- invertImage(ref.roi$ROI)
}

plot(ref.roi$ROI)
```

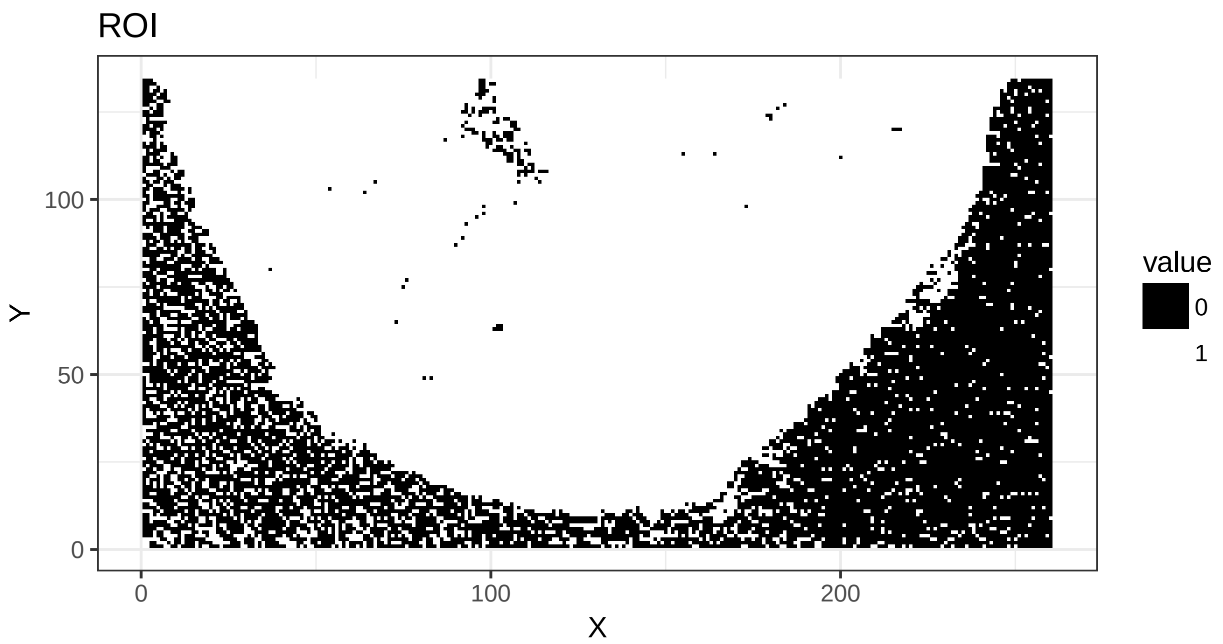

It is important to select the best reference image and ROI mask parameters, since they will be used for the following filtering steps.

Global similarity filter

The `globalPeaksFilter` is applied on the intensity matrix. This filter selects all the peaks signals that are similar with the provided reference image. A threshold of 0 for the Pearson's correlation guarantees that only the peaks signals present in the ROI are considered for further analysis. Usually, this filter drastically reduces the number of matched peaks, since several noisy peaks are often localized outside the ROI. Here, we select the peaks signals with a SSIM value with the reference image larger than 0. It is important to notice that for consistency with the correlation measures, both the SSIM and NMI are scaled in [-1, 1].

```
## Select peaks based on correlation with ROI mask. When comparing with the ROI,
## use "spearman"
start.time <- Sys.time()

sel.peaks <- globalPeaksFilter(msiData = msiX,
                               referenceImage = ref.roi$ROI,
                               method = "spearman",
                               threshold = 0,
                               verbose = TRUE)
```

```
## Calculating the similarity values...
```

```
Sys.time() - start.time
```

```
## Time difference of 6.866593 secs
```

```r
## Apply the filter
msiX <- applyPeaksFilter(msiX, sel.peaks)
cat("new dimensions:", dim(getIntensityMat(msiX)), "\n")
```

```
## new dimensions: 34840 653
```

Generally, `globalPeaksFilter` reduces significantly the number of matched peaks and increases the contrast between the ROI and the region outside.

Here, we update the reference image and ROI after applying the filter. Usually, after applying `globalPeaksFilter`, the TIC image captures the real signal very well. For this reason, also Otsu's thresholding can be applied.

Connected pixels count based filter

`countPixelsFilter` is applied to remove peaks localized in small or scattered regions. This filter removes all the peaks which intensities are localized in connected regions smallest than `minNumPixels` within the ROI. Different aggressiveness levels determine whether also the connected components outside the ROI should be evaluated. Pixel size is equal to about 10um Here, we are interested in all the sub-regions with an area larger than 400umˆ2. Different levels of aggressiveness can produce different results, as shown here.

For high resolution images, it is more likely to find meaningful signal that appears to be scattered. For this reason, we reduce the scatteredness applying a morphological closing before counting the connected regions, setting `closePeakImage = TRUE`. By default the closing kernel size is equal to 5 pixels.

We will set `aggressive` equal to 1.

```r
start.time <- Sys.time()
res.count.filter <- countPixelsFilter(msiData = msiX,
                                      roiImage = ref.roi$ROI,
                                      minNumPixels = 4,
                                      closePeakImage = TRUE,
                                      aggressive = 1,
                                      verbose = FALSE)
Sys.time() - start.time
```

```
## Time difference of 19.68534 secs
```

```r
print(length(res.count.filter$sel.peaks))
```

```
## [1] 339
```

```r
## Apply the filter - the second element of the list corresponds to aggressive = 1
msiX <- applyPeaksFilter(msiX, res.count.filter)
```

Complete spatial randomness filter

A filter based on testing for complete spatial randomness (CSR) is applied on the spatial distribution of the binarized peaks signals. Here we apply the Kolmogorov-Smirnov `"KS"` to check whether the pixel distribution follows the density of the covariate (calculated, in this case, as the smoothed TIC image). The Bonferroni-corrected p-values are thresholded to 0.001 in order to select the non-randomly scattered peaks.

```r
res.csr.stats <- CSRPeaksFilter(msiData = msiX,
                                method = "KS",
                                covMethod = "sum",       # --------------------
                                useFullMZCov = TRUE,     # Covariate arguments
```

```r
                                  smoothCov = TRUE,         #
                                  invertCov = FALSE,        #----------------------
                                  adjMethod = "bonferroni",
                                  returnQvalues = TRUE,
                                  plotCovariate = FALSE,
                                  verbose = TRUE)
```

```
## Calculating the reference image. This may take a while...
## Scaling all the peaks intensities in [0, 1]
## Starting CSR tests. This may take a while...
```

```r
cat(sum(res.csr.stats$p.value < 0.001), "\n")
```

```
## 227
```

```r
cat(sum(res.csr.stats$q.value < 0.001), "\n")
```

```
## 205
```

Apply the filtering

```r
csr.filt <- createPeaksFilter(which(res.csr.stats$q.value < 0.001))

msiX <- applyPeaksFilter(msiX, csr.filt)
```

Compare the original and filtered TIC images.

```r
im <- matrix(apply(maldiData, 1, sum),
             getShapeMSI(msiX)[1], getShapeMSI(msiX)[2]) # Original intensities
im.filt <- matrix(apply(getIntensityMat(msiX), 1, sum),
                  getShapeMSI(msiX)[1], getShapeMSI(msiX)[2]) # Filtered
plot(msImage(im, name = "TIC original"))
```

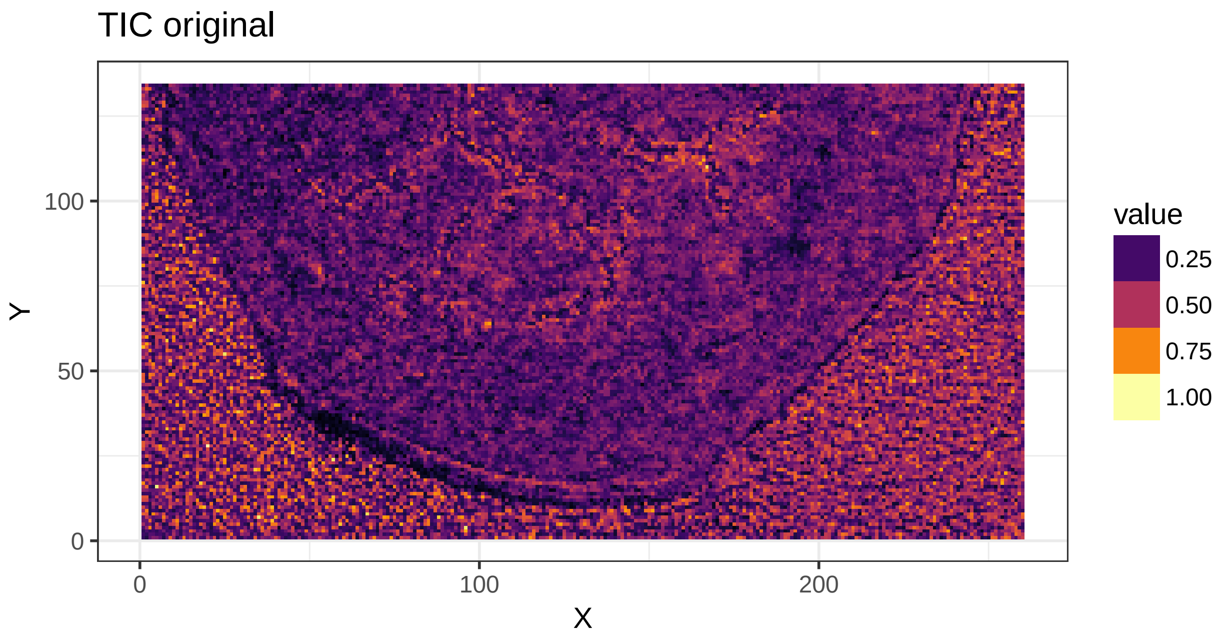


```
plot(msImage(im.filt, name = "TIC filtered"))
```

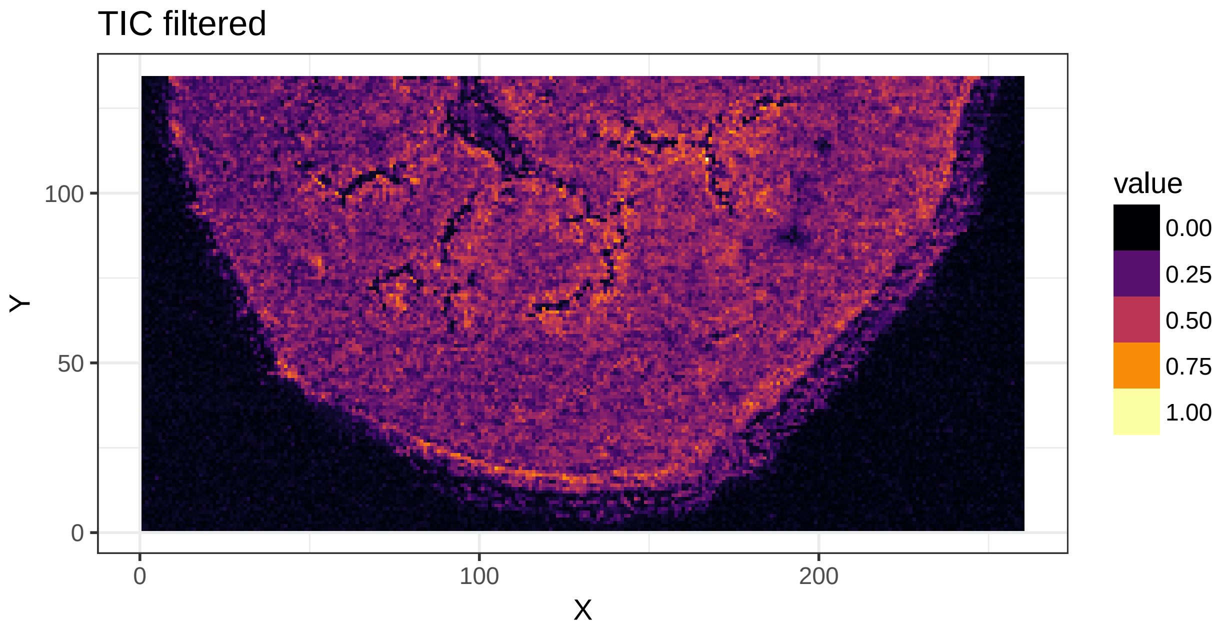

Comparison between the original and filtered datasets

Here we extract the first 3 principal components and visualize them as RGB channels. The scores are first scaled in [0, 1].

```
## PCA original dataset
start.time <- Sys.time()
pca.orig <- prcomp(maldiData)
Sys.time() - start.time
```

```
## Time difference of 2.220225 mins
```

```
## PCA filtered dataset
start.time <- Sys.time()
pca.filt <- prcomp(getIntensityMat(msiX))
Sys.time() - start.time
```

```
## Time difference of 4.407205 secs
```

```
## Plot the first 3 principal components as RGB channels
im.orig.rgb <- array(NA, c(sz[1], sz[2], 1, 3))
for (i in 1:3) {
  im.orig.rgb[, , 1, i] <- matrix((pca.orig$x[, i] - min(pca.orig$x[, i])) /
                                    (max(pca.orig$x[, i]) - min(pca.orig$x[, i])),
                                  sz[1], sz[2])[, sz[2]:1]
}
im.filt.rgb <- array(NA, c(sz[1], sz[2], 1, 3))
```

```r
for (i in 1:3) {
  im.filt.rgb[, , 1, i] <- matrix((pca.filt$x[, i] - min(pca.filt$x[, i])) /
                                    (max(pca.filt$x[, i]) - min(pca.filt$x[, i])),
                                  sz[1], sz[2])[, sz[2]:1]
}
par(mfrow = c(1, 2), mar = c(1, 0.2, 1, 0.2))
plot(as.cimg(im.orig.rgb), main = "First 3 PC, original data")
plot(as.cimg(im.filt.rgb), main = "First 3 PC, filtered data")
```

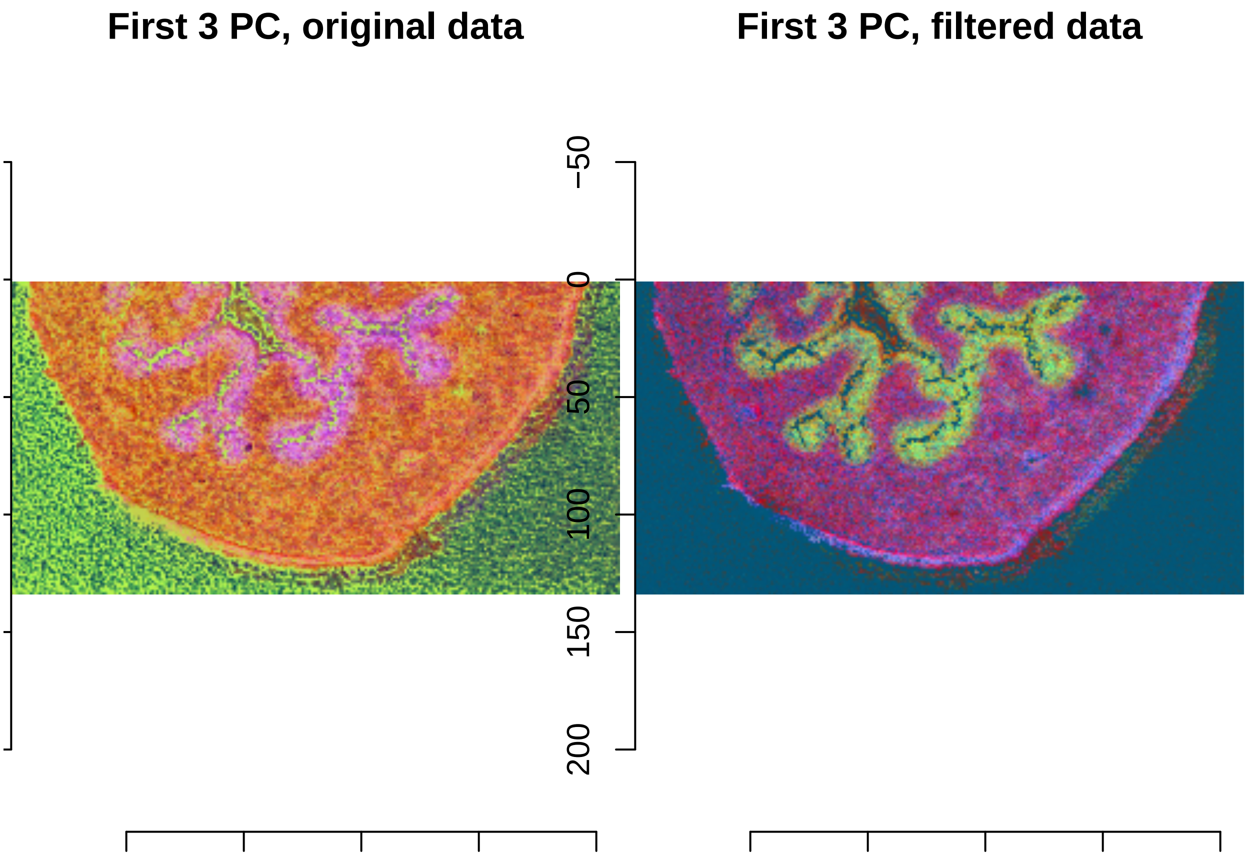

Here we compare the clustering results using k-means with a number of clusters equal to 4 on the first K principal components scores responsible of the 95% of the total variance.

```r
## Determine the number of principal components responsible of the 95% of the
## total variance
var.perc.orig <- cumsum(pca.orig$sdev^2 / sum(pca.orig$sdev^2))
var.perc.filt <- cumsum(pca.filt$sdev^2 / sum(pca.filt$sdev^2))
num.comps.orig <- which(var.perc.orig >= 0.95)[1]
num.comps.filt <- which(var.perc.filt >= 0.95)[1]
cat("Num. components original dataset =", num.comps.orig, "\n")
```

```
## Num. components original dataset = 166
```

```r
cat("Num. components filtered dataset =", num.comps.filt, "\n")
```

```
## Num. components filtered dataset = 74
```

```r
start.time <- Sys.time()
clust.orig <- kmeans(pca.orig$x[, 1:num.comps.orig], centers = 4,
```

```r
                     iter.max = 1000, nstart = 50)
Sys.time() - start.time
```

```
## Time difference of 29.45617 secs
```

```r
start.time <- Sys.time()
clust.filt <- kmeans(pca.filt$x[, 1:num.comps.filt], centers = 4,
                     iter.max = 1000, nstart = 50)
```

```
## Warning: Quick-TRANSfer stage steps exceeded maximum (= 1742000)
```

```r
Sys.time() - start.time
```

```
## Time difference of 3.415488 secs
```

```r
## Visualize the clusters
par(mfrow = c(1, 2), mar = c(1, 0.2, 1, 0.2))

clust.img.orig <- matrix(clust.orig$cluster, sz[1], sz[2])
clust.img.filt <- matrix(clust.filt$cluster, sz[1], sz[2])

plot(msImage(clust.img.orig, name = "K-means orig. dataset"))
```

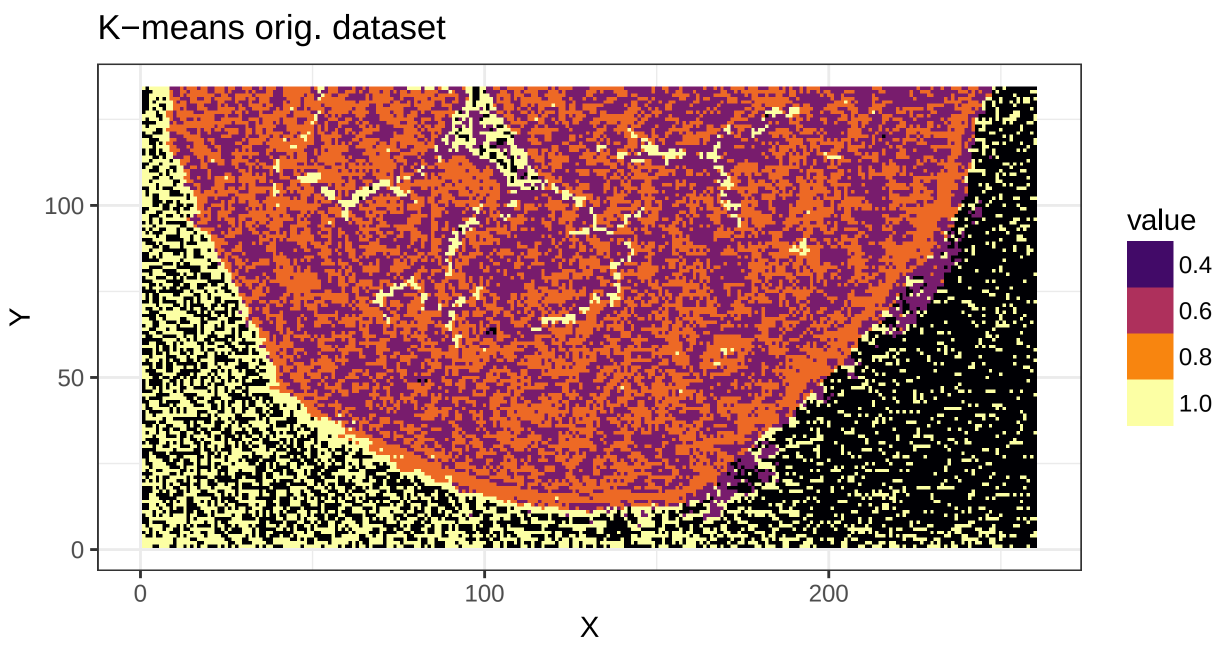


```r
plot(msImage(clust.img.filt, name = "K-means filt. dataset"))
```

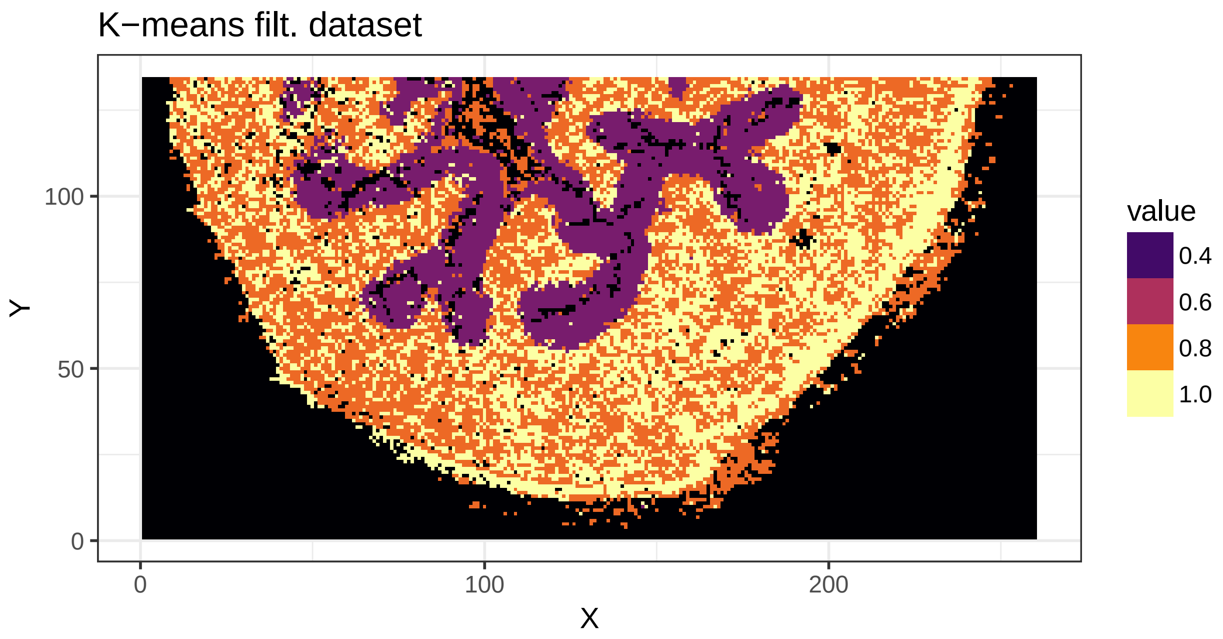

Finally, let's check the image corresponding to the smallest number of signal pixels.

```
num.pixels <- nrow(getIntensityMat(msiX))
frac <- array(0, ncol(getIntensityMat(msiX)))
for (i in 1:length(frac))
{
  frac[i] <- sum(getIntensityMat(msiX)[, i] != 0) / num.pixels
}

cat("num. peaks with freq. less than 1% =", sum(frac < 0.01), "\n")
```

```
## num. peaks with freq. less than 1% = 40
```

```
## Plot the spatial distribution an example peak with a fraction smaller than 1%
small.signals <- which(frac < 0.01)
small.idx <- 4
print(frac[small.idx])
```

```
## [1] 0.009041332
```

```
mz.value <- round(getMZ(msiX)[small.idx], 4)
im.smallest <- matrix(getIntensityMat(msiX)[, small.idx],
                      getShapeMSI(msiX)[1], getShapeMSI(msiX)[2])

plot(msImage(im.smallest, name = as.character(mz.value)))
```

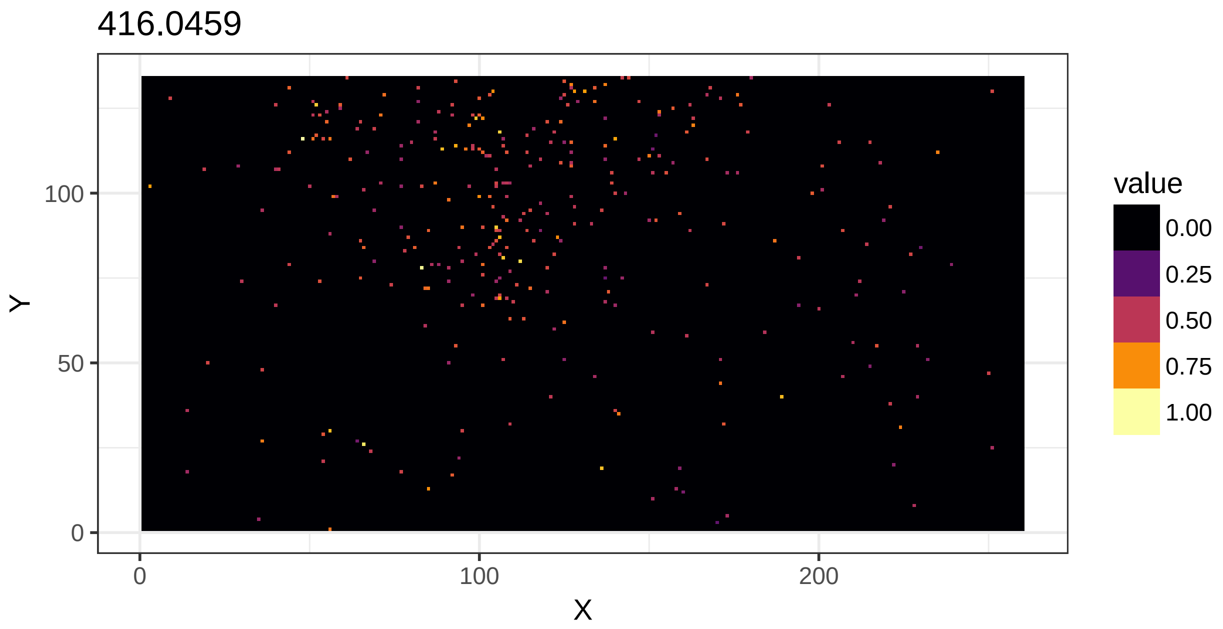

Clearly, this intensity image shows a non-random pattern that would have been removed by a naive fraction filter with a threshold of 1%.

Comparison with the selected peaks in Figure 1-A found in (Römpp et al. 2010)

```
## Selected m/z values used in Figure 1-A of RÃ¶mpp et al. 2010. Probably, the
## reported peak 743.5482 m/z is the isotopic form of the significant peak 742.5322 m/z
sel.mz <- c(741.5318, 798.5408, 742.5322)
sel.idx <- which(round(getMZ(msiX), 4) %in% sel.mz)
sel.idx <- c(sel.idx[2], sel.idx[3], sel.idx[1])

im.rompp.rgb <- array(NA, c(sz[1], sz[2], 1, 3))
for (i in 1:3) {
  tmp <- getIntensityMat(msiX)[, sel.idx[i]]
  im.rompp.rgb[, , 1, i] <- matrix(tmp / max(tmp),
                                   sz[1], sz[2])
}
plot(as.cimg(im.rompp.rgb), main = "Reconstruction of Figure 1-A from RÃ¶mpp et al. 2010")
```

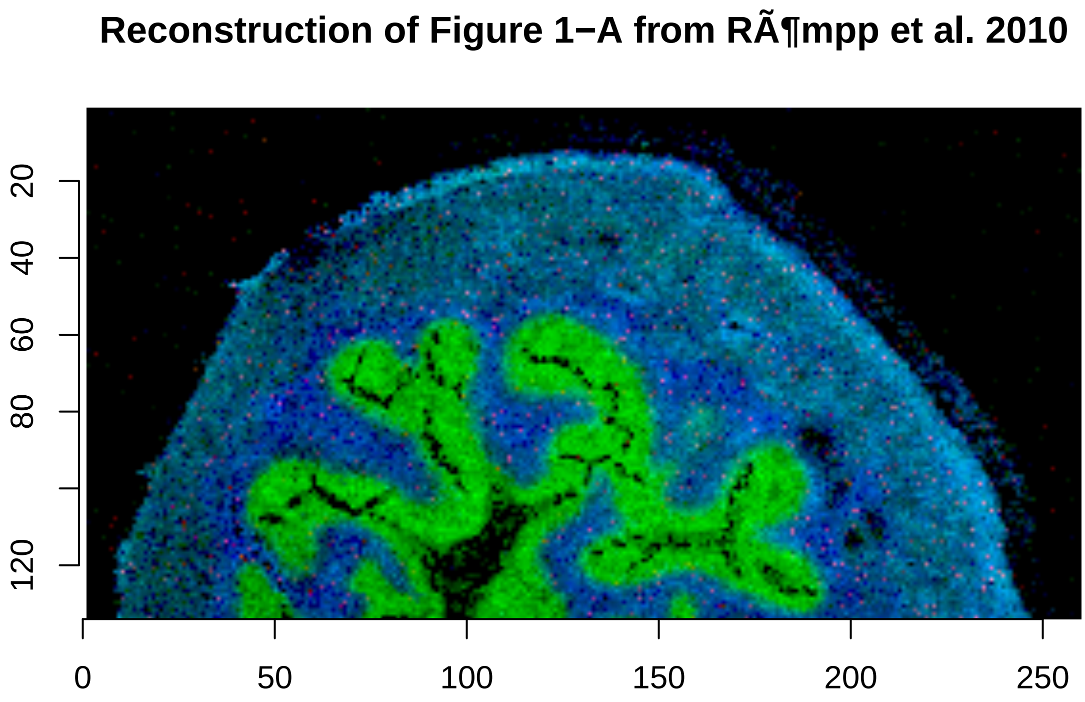

Comparison with the selected peaks in Figure 1-B found in (Römpp et al. 2010)

```
## Selected m/z values used in Figure 1-B of RÃ¶mpp et al. 2015.
sel.mz <- c(616.1128, 798.5408, 812.5564)
sel.idx <- which(round(getMZ(msiX), 4) %in% sel.mz)
sel.idx <- c(sel.idx[1], sel.idx[3], sel.idx[2])

im.rompp.rgb <- array(NA, c(sz[1], sz[2], 1, 3))
for (i in 1:3) {
  tmp <- getIntensityMat(msiX)[, sel.idx[i]]
  im.rompp.rgb[, , 1, i] <- matrix(tmp / max(tmp),
                                   sz[1], sz[2])
}
plot(as.cimg(im.rompp.rgb), main = "Reconstruction of Figure 1-B from RÃ¶mpp et al. 2010")
```

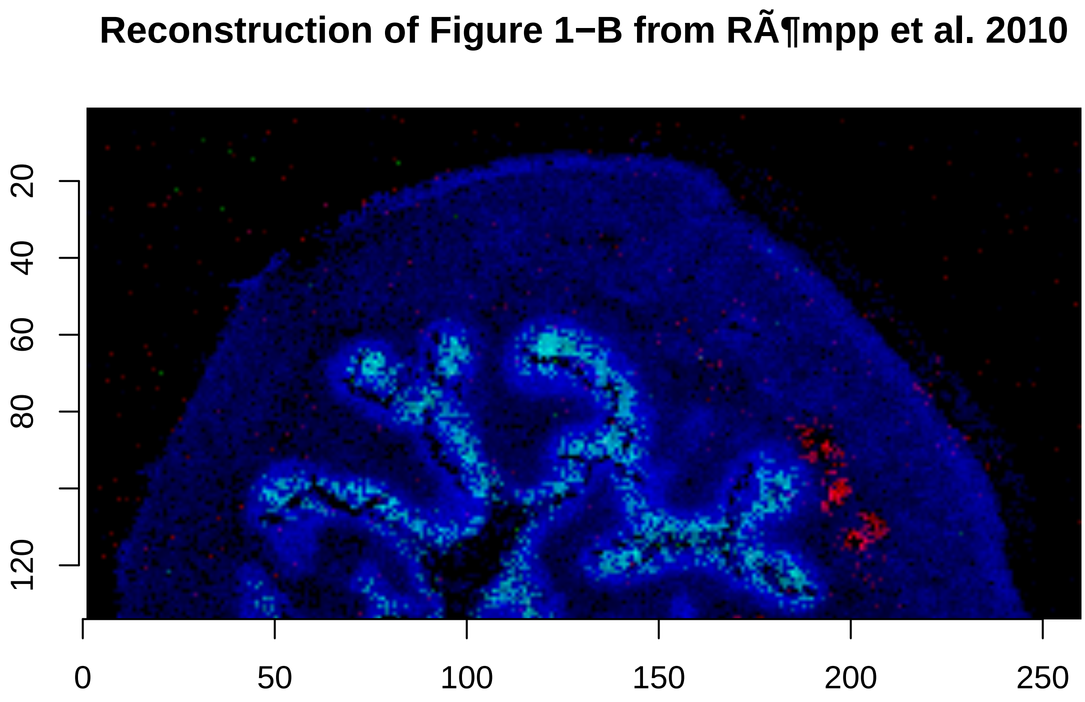

Verify selected peaks

Plot the peaks average intensities and the selected peaks.

```
## Calculate the average signal
maldiData[is.na(maldiData)] <- 0
mzOriginal <- as.numeric(attr(maldiData, 'mass'))
mzNew <- getMZ(msiX)
avgOriginal <- apply(maldiData, 2, mean)

newIdx <- which(mzOriginal %in% mzNew)
filteredIdx <- which(!(mzOriginal %in% mzNew))
stopifnot(length(newIdx) + length(filteredIdx) == length(mzOriginal))
```

```
plot(mzOriginal, avgOriginal, col = "black", pch = 4, type = "h", xlab = 'm/z',
     ylab = 'average intensity')
points(mzNew, avgOriginal[newIdx], col = "red", cex = 0.5)
```

Plot the top 10 filtered peaks images.

```
## Sort the filtered peaks by their average intensity and take the top 10
sortedFilterIdx <- sort(avgOriginal[filteredIdx], decreasing = T, index.return = T)$ix[1:10]

par(mfrow = c(2, 5), mar = c(2, 2, 1, 0.5))
for (i in 1:length(sortedFilterIdx))
{
```

```
  im <- log(maldiData[, filteredIdx[sortedFilterIdx[i]]] + 1)
  im <- im / max(im)
  im <- matrix(im, sz[1], sz[2])
  im[is.na(im)] <- 0
  ## Plot as yellow/black image
  im.rgb <- array(0, c(sz[1], sz[2], 1, 3))
  im.rgb[, , 1, 1] <- im
  im.rgb[, , 1, 2] <- im
  plot(as.cimg(im.rgb), main = round(mzOriginal[filteredIdx[sortedFilterIdx[i]]], 4))
}
```

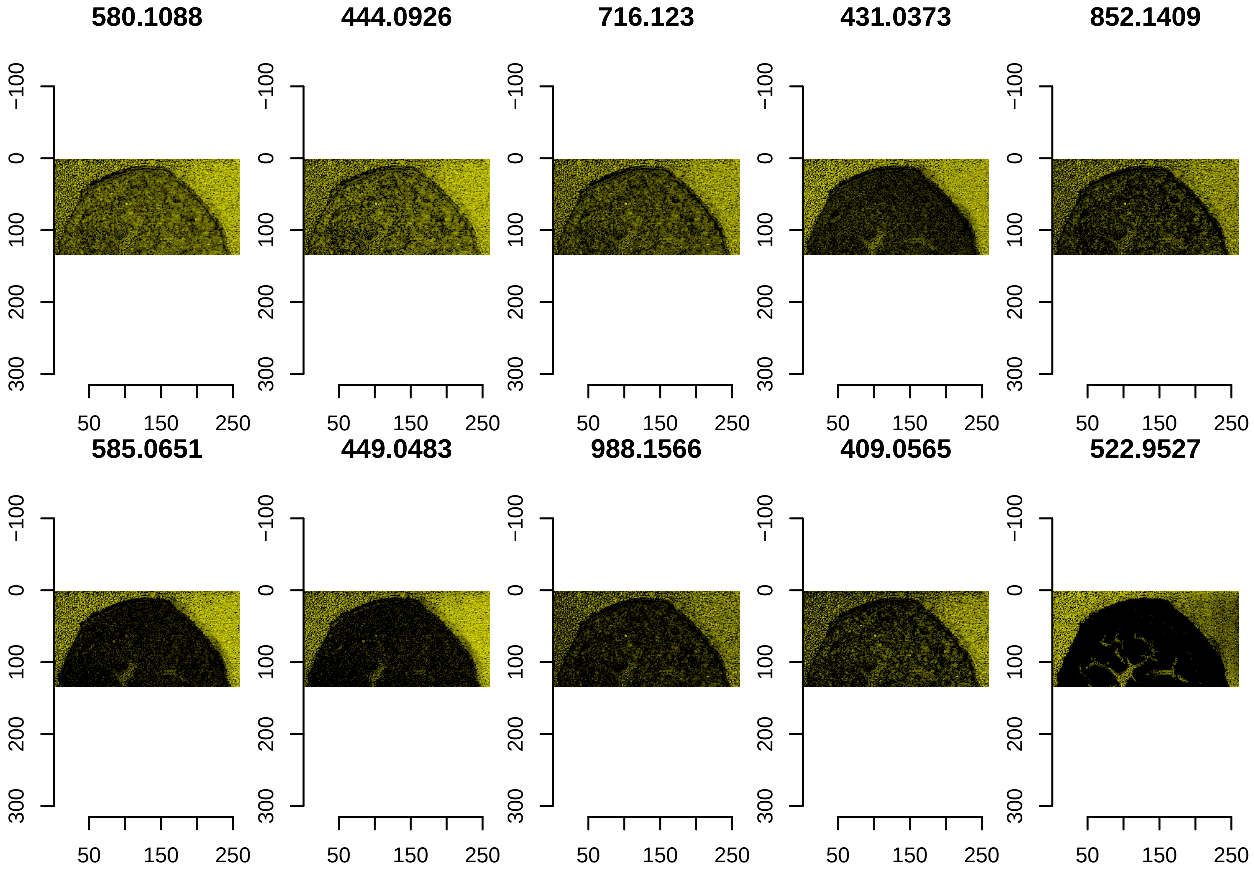

Plot the top 10 retained peaks images.

```
sortedNewIdx <- sort(avgOriginal[newIdx], decreasing = T, index.return = T)$ix[1:10]

par(mfrow = c(2, 5), mar = c(2, 2, 1, 0.5))
for (i in 1:length(sortedNewIdx))
{
  im <- log(maldiData[, newIdx[sortedNewIdx[i]]] + 1)
  im <- im / max(im)
  im <- matrix(im, sz[1], sz[2])
  im[is.na(im)] <- 0
  ## Plot as yellow/black image
  im.rgb <- array(0, c(sz[1], sz[2], 1, 3))
  im.rgb[, , 1, 1] <- im
  im.rgb[, , 1, 2] <- im
```

```
  plot(as.cimg(im.rgb), main = round(mzOriginal[newIdx[sortedNewIdx[i]]], 4))
}
```

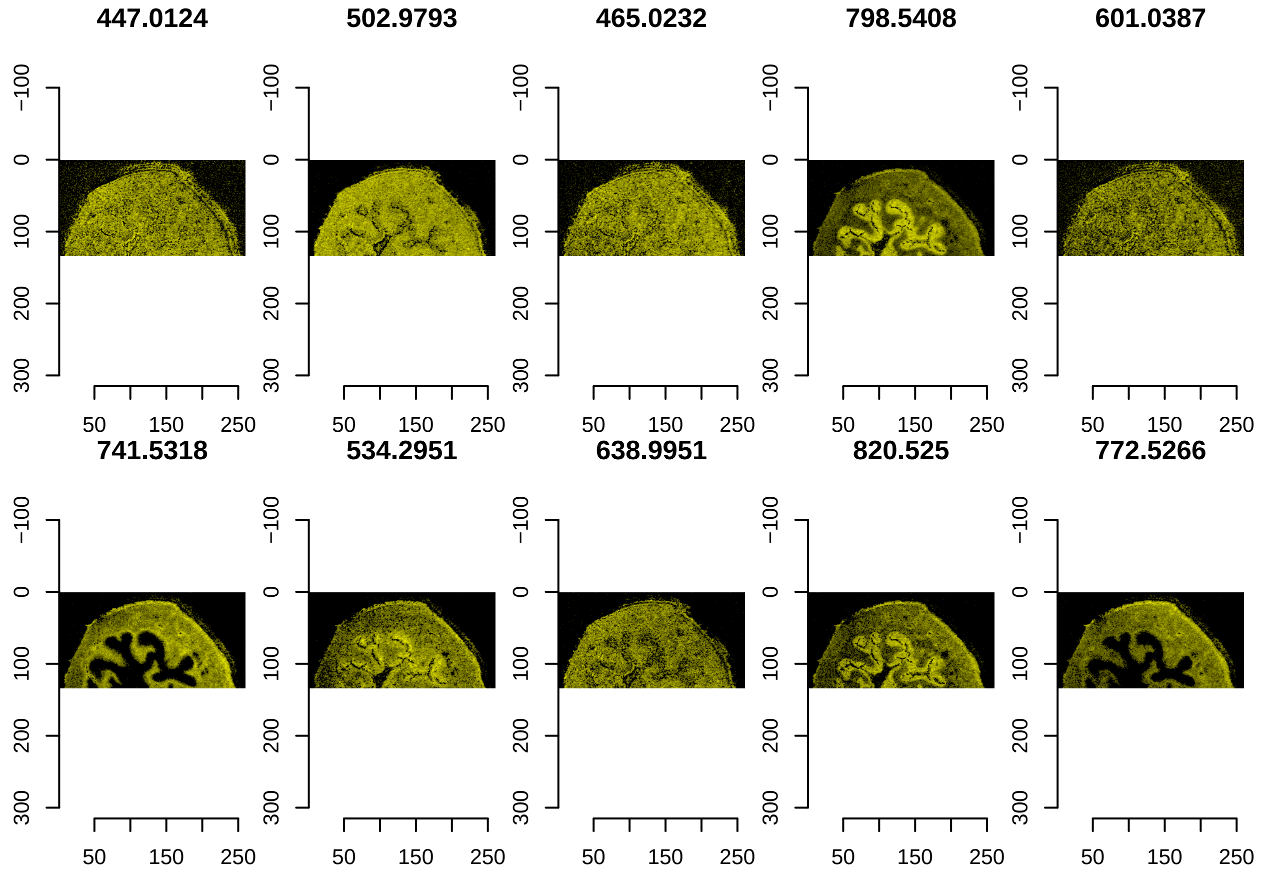

References

Gibb, Sebastian, and Korbinian Strimmer. 2012. "MALDIquant: A Versatile R Package for the Analysis of Mass Spectrometry Data." *Bioinformatics* 28 (17). Oxford University Press: 2270–1.

Römpp, Andreas, Sabine Guenther, Yvonne Schober, Oliver Schulz, Zoltan Takats, Wolfgang Kummer, and Bernhard Spengler. 2010. "Histology by Mass Spectrometry: Label-Free Tissue Characterization Obtained from High-Accuracy Bioanalytical Imaging." *Angewandte Chemie International Edition* 49 (22). WILEY-VCH Verlag: 3834–8. doi:10.1002/anie.200905559.
