# Supplementary material for: SPUTNIK: an R package for filtering of spatially related peaks in mass spectrometry imaging data
Source: Bioinformatics. 2018 Jul 13;35(1):178–80. doi: 10.1093/bioinformatics/bty622 (PMC6298046; doi:10.1093/bioinformatics/bty622)
Supplement: Supplementary Data 3 [file bty622_supplementary_data_3.pdf]

SPUTNIK DESI-MSI workflow

Contents

Introduce an artificial split peak at 855.6744 m/z . . . 2
Merge estimated split peaks . . . 2
Determine the reference image and the ROI . . . 6
Global similarity filter . . . 8
Connected pixels count based filter . . . 11
Complete spatial randomness filter . . . 12
Comparison between the original and filtered datasets . . . 12
References . . . 16

```
library("SPUTNIK")
library("imager")
```

```
## Loading required package: plyr

## Loading required package: magrittr

##
## Attaching package: 'imager'

## The following object is masked from 'package:magrittr':
##
##     add

## The following object is masked from 'package:plyr':
##
##     liply

## The following objects are masked from 'package:stats':
##
##     convolve, spectrum

## The following object is masked from 'package:graphics':
##
##     frame

## The following object is masked from 'package:base':
##
##     save.image
```

IMPORTANT! Do not normalize the peaks intensity matrix before running the `splitPeaksFilter`. Also the variance stabilizing transformation should be applied after the filter. The ovarian cancer DESI-MSI data set acquired with XEVO Q-TOF in negative ion mode has been pre-processed using [MALDIquant](https://github.com/cran/MALDIquant) (Gibb and Strimmer 2012). No filter was applied during the pre-processing. The intensity matrix has been loaded with the variable name `desiData`. The number of pixels is equal to 73 x 118 (x, y). MALDIquant assigns a NA to empty peak signals, these should be converted into zeros before running the analysis.

```
desiData <- ovarianDESIDoria2016()
```

```
## Downloading the data from the repository...
## Loading the data in the R environment...
```

```
desiData[is.na(desiData)] <- 0
```

Define the geometrical shape variable, `sz`.

```
sz <- attr(desiData, "size")
```

Introduce an artificial split peak at 855.6744 m/z

```
mz <- as.numeric(colnames(desiData))

## Select the peaks with largest number of signal peaks
num_pix <- array(NA, ncol(desiData))
for (i in 1:ncol(desiData))
{
  num_pix[i] <- sum(desiData[, i] != 0) / prod(sz)
}

## Select 885 m/z
sel_peak <- 1219
print(mz[sel_peak])
```

```
## [1] 885.6744
```

```
## Split the selected peaks
split_mz <- mz[sel_peak] + 0.001
stopifnot(!split_mz %in% mz) ## Check if introducing duplicated m/z values

new_intensity <- matrix(0, nrow(desiData), length(split_mz),
                        dimnames = list(NULL, split_mz))
## Select randomly 30% of pixels and assign their intensity to the new mass
idx_split_pixels <- sample(nrow(desiData), round(0.3 * nrow(desiData)))
new_intensity[idx_split_pixels, 1] <- desiData[idx_split_pixels, sel_peak]
desiData[idx_split_pixels, sel_peak] <- 0

desiData <- cbind(desiData, new_intensity)
desiData <- desiData[, sort(as.numeric(colnames(desiData)), index.return = T)$ix]
```

```
## Assign the NA values to 0 and print the dimensions of the intensity matrix
print(dim(desiData))
```

```
## [1] 8614 1376
```

```
# Create the MSI dataset. MALDIquant assigns the matched m/z values to the
# columns name of the intensity matrix
msiX <- msiDataset(values = desiData,
                   mz = as.numeric(colnames(desiData)),
                   rsize = sz[1], csize = sz[2])
```

Merge estimated split peaks

Firstly, we test whether some peaks have been split during the matching process. This can be due to issues with the spectra alignment. Usually, this error generates close peaks that are spatially localized in complementary regions. In the `splitPeaksFilter`, the smallest distance that defines close peaks is expressed in ppm. This value should be carefully chosen to be close to the instrumental error; a too large value will produce a wrong merging. The `sharedPixelsRatio` represents the maximum accepted ratio between the pixels shared by two or more peaks and the total number of peaks signal pixels.

Show that 885.6744 m/z is split.

```
ion_image_1 <- msImage(matrix(msiX@matrix[, 1219], msiX@nrow, msiX@ncol),
                      name = as.character(round(msiX@mz[1219], 4)), scale = T)
plot(ion_image_1)
```

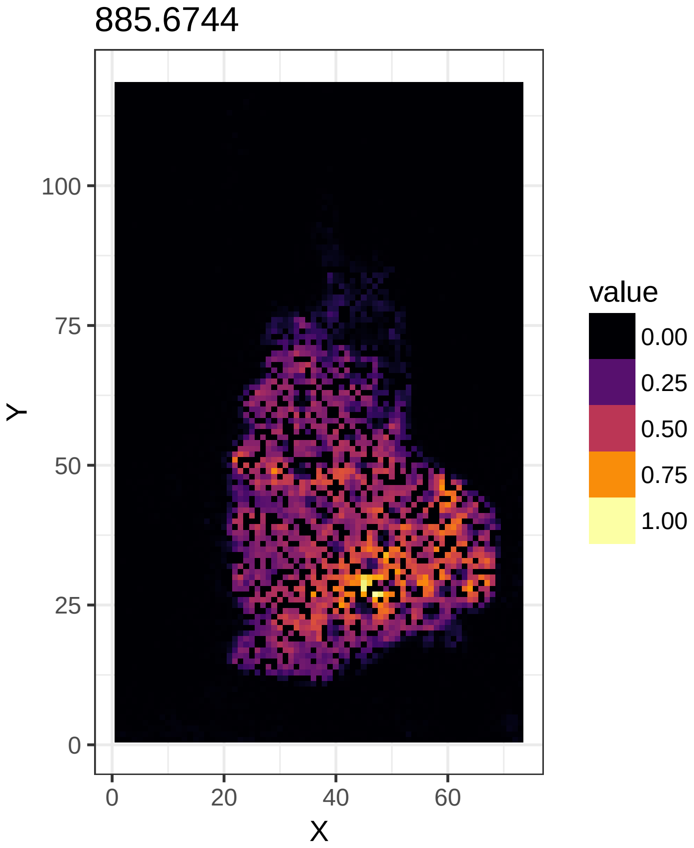


```
ion_image_2 <- msImage(matrix(msiX@matrix[, 1220], msiX@nrow, msiX@ncol),
                      name = as.character(round(msiX@mz[1220], 4)), scale = T)
plot(ion_image_2)
```

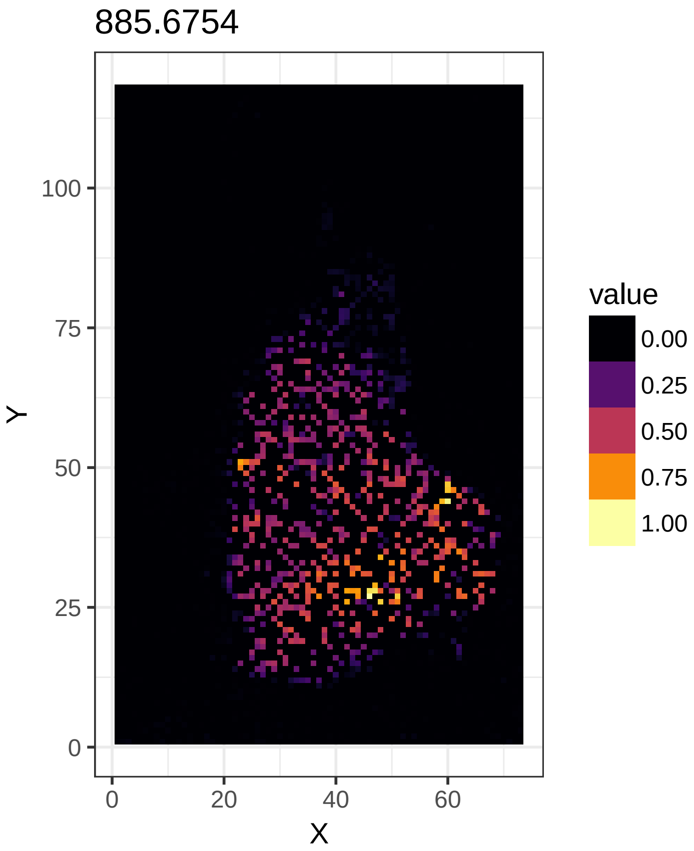

Estimate the split peaks.

```
split.peaks <- splitPeaksFilter(msiData = msiX,
                                mzTolerance = 10,
                                sharedPixelsRatio = 0,
                                sparseness = "scatter.ratio",
                                threshold = 0.5,
                                returnDetails = T,
                                verbose = T)
```

```
## Determining close peaks. This may take a while...
## Min. distance between peaks (ppm) = 1.129083
## Found 1 groups of peaks to test for scatteredness.
## Testing scatteredness of merged images...
## 2 peaks merged in 1 peaks.
```

Apply the filter to the dataset.

```
msiX <- applyPeaksFilter(msiX, split.peaks)
```

Plot the reconstructed image.

```
ion_image <- msImage(matrix(msiX@matrix[, 1219], msiX@nrow, msiX@ncol),
                     name = as.character(round(mz[sel_peak], 4)), scale = T)
plot(ion_image)
```

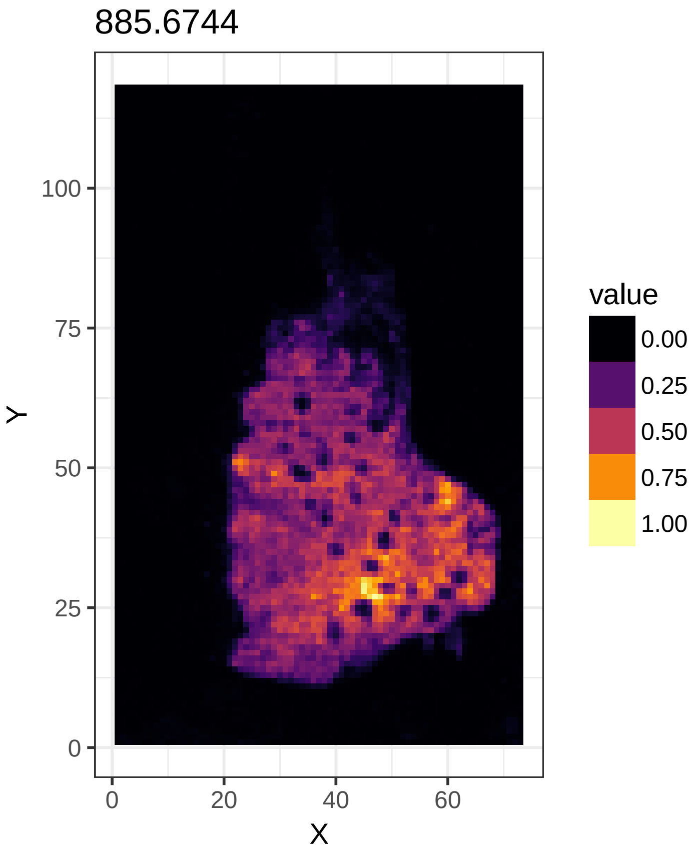

Now we can normalize the intensities with median scaling and apply the log-transformation. This was skipped during `MALDIquant` pre-processing in order to have a correct normalization after the peaks merging. Let's apply the transformations also to the original matrix for a subsequent comparison of the results.

```
## Normalization and log-transformation of the original intensity matrix.
Start.time <- Sys.time()

## Apply median scaling without considering the zeros.
desiData[desiData == 0] <- NA

for (i in 1:nrow(desiData))
{
  desiData[i, ] <- desiData[i, ] /
    median(desiData[i, ], na.rm = T)
}

desiData[is.na(desiData)] <- 0

## Apply the log-transformation adding a constant equal to 1 in order to preserve
## the zeros
desiData <- log(desiData + 1)

Sys.time() - Start.time
```

```
## Time difference of 1.557799 secs
```

Normalize the msiX matrix elements.

```r
## Apply median scaling and log-transformation to the MSI dataset
start.time <- Sys.time()

msiX <- normIntensity(msiX, "median")
msiX <- varTransform(msiX, "log")

Sys.time() - start.time
```

```
## Time difference of 1.578774 secs
```

Determine the reference image and the ROI

Before, extracting the reference image and the ROI, we to select the measure to be used to calculate the reference intensities among `sum`, `mean`, `median`, and `pca`. Furthermore, we have to provide the method used to generate the ROI, among `otsu` and `kmeans`.

```r
## Now apply Gaussian smoothing (standard deviation = 2, by default) to the
## reference image, before calculating the binary ROI mask with the Otsu's
## thresholding.

ref.roi <- refAndROIimages(msiData = msiX,
                           refMethod = "sum",
                           roiMethod = "otsu",
                           mzQueryRef = numeric(),
                           useFullMZRef = TRUE,
                           smoothRef = TRUE, # <== SMOOTHING
                           invertRef = FALSE,
                           verbose = TRUE)
```

```r
## Visualize the reference image. Now it appears smoothed.
plot(ref.roi$Reference)
```

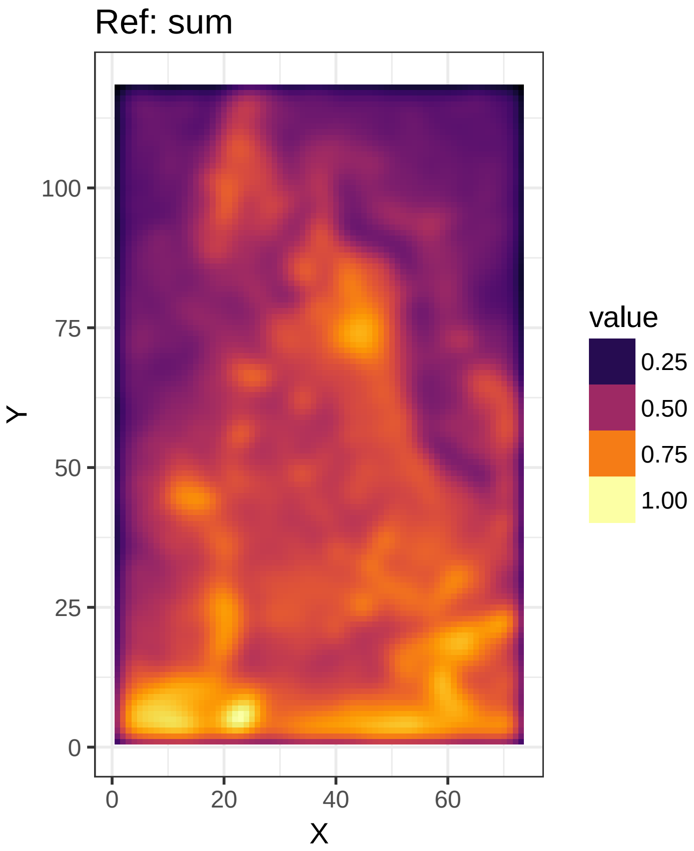


```
## Visualize the binary ROI mask. The smoothing has reduces the pixelation of
## the binary mask.
plot(ref.roi$ROI)
```

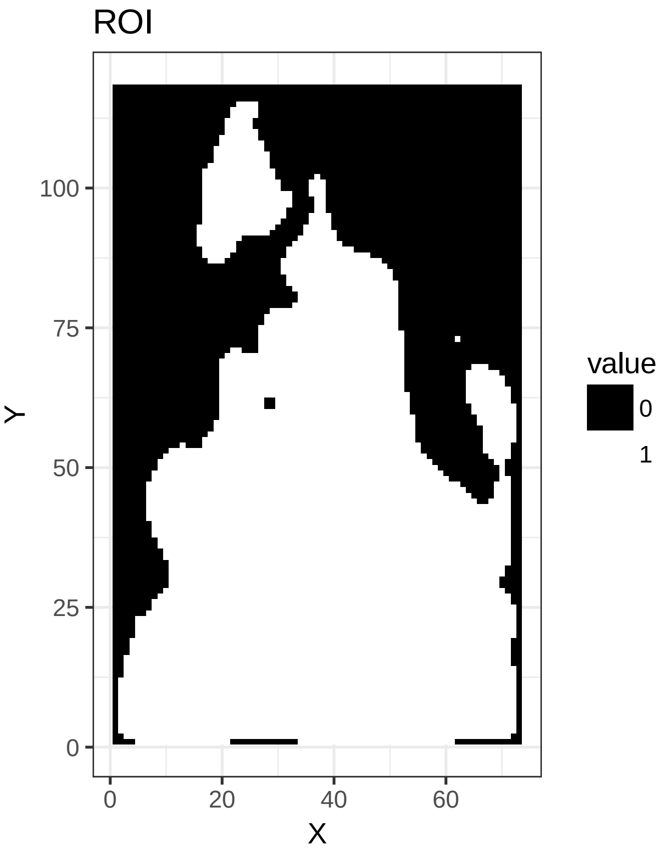

The ROI mask is not pixelated.

It is important to select the best reference image and ROI mask parameters, since they will be used for the following filtering steps.

Global similarity filter

The `globalPeaksFilter` is applied on the intensity matrix. This filter selects all the peaks signals that are similar with the provided reference image. A threshold of 0 for the Pearson's correlation guarantees that only the peaks signals present in the ROI are considered for further analysis. Usually, this filter drastically reduces the number of matched peaks, since several noisy peaks are often localized outside the ROI. Here, we select the peaks signals with a SSIM value with the reference image larger than 0. It is important to notice that for consistency with the correlation measures, both the SSIM and NMI are scaled in [-1, 1].

```
## globalPeaksFilter selects the peaks which intensities are similar more than
## "threshold" with the "referenceImage". In this case, the referenceImage is
## the smoothed sum of peaks intensities, calculated previously, the selected
## peaks have Pearson's correlation with it larger than 0. Also, the binary
## ROI mask can be used as referenceImage, but in that case it is suggested
## to use Spearman's correlation, SSIM or NMI.
start.time <- Sys.time()

sel.peaks <- globalPeaksFilter(msiData = msiX,
                               referenceImage = ref.roi$Reference,
                               method = "pearson",
                               threshold = 0,
```

```r
                                 verbose = TRUE)
```

```
## Calculating the similarity values...
```

```r
Sys.time() - start.time
```

```
## Time difference of 0.2483621 secs
```

```r
## Apply the filter to the MSI dataset
msiX <- applyPeaksFilter(msiX, sel.peaks)
cat("new dimensions:", dim(getIntensityMat(msiX)), "\n")
```

```
## new dimensions: 8614 985
```

Generally, `globalPeaksFilter` reduces significantly the number of matched peaks and increases the contrast between the ROI and the region outside.

Here, we update the reference image and ROI after applying the filter.

```r
## Re-calculate the reference and ROI mask after applying the correlation based
## filter. After removing the peaks localized outside the region occupied by the
## tissue section, the reference and ROI become much clearer. Here we use the
## same parameters used previously.
ref.roi <- refAndROIimages(msiData = msiX,
                           refMethod = "sum",
                           roiMethod = "otsu",
                           useFullMZRef = TRUE,
                           smoothRef = FALSE,
                           invertRef = FALSE,
                           verbose = TRUE)
```

Compare the original and the filtered TIC images.

```r
## Visualize the sum of peaks intensities image from the original and filtered
## dataset.
par(mfrow = c(1, 2), mar = c(1, 0.2, 1, 0.2))
im <- apply(desiData, 1, sum)

plot(msImage(values = matrix(im, getShapeMSI(msiX)[1], getShapeMSI(msiX)[2]),
             name = "TIC original", scale = T))
```

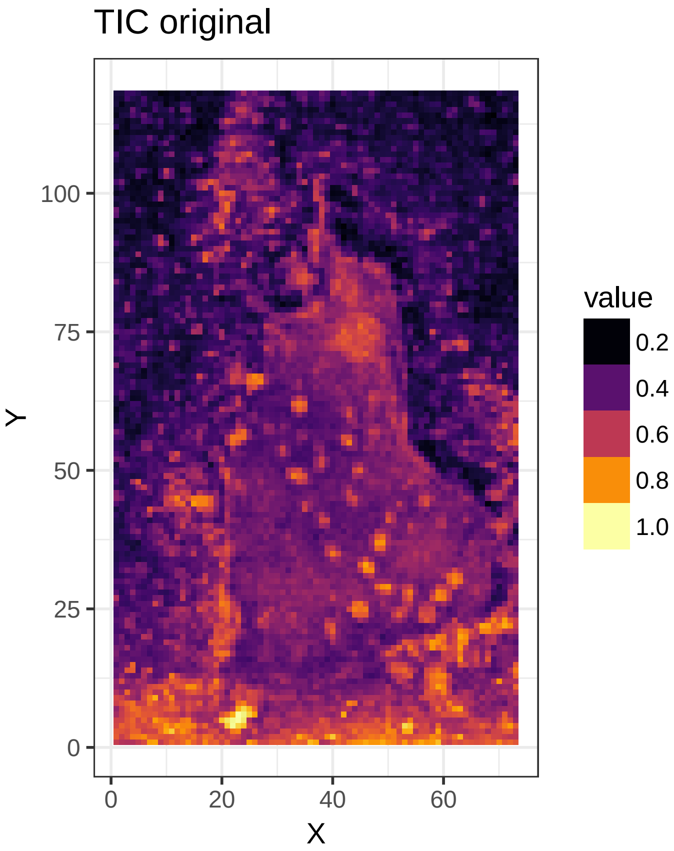


```
plot(ref.roi$Reference)
```

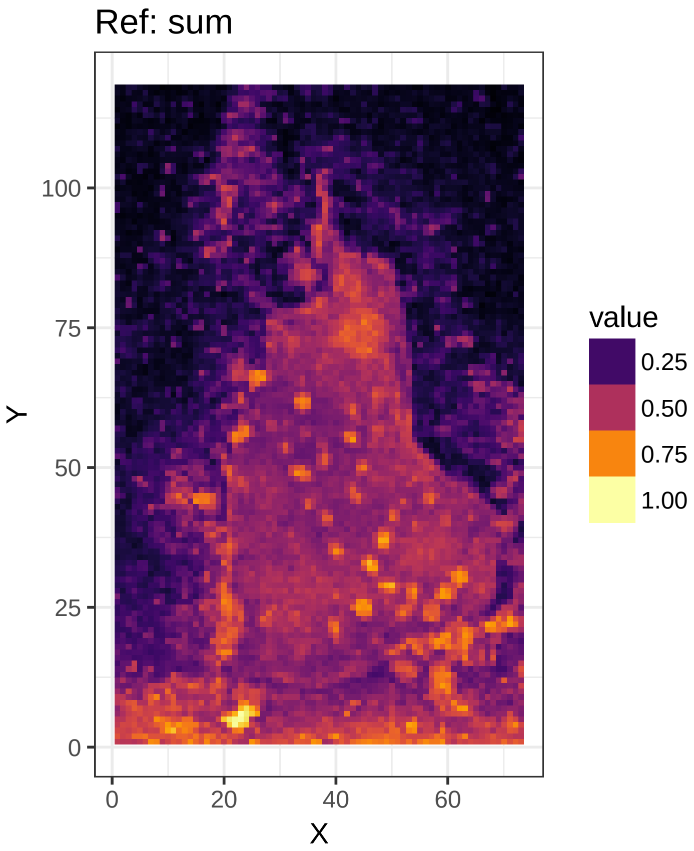

Connected pixels count based filter

`countPixelsFilter` is applied to remove peaks localized in small or scattered regions. This filter removes all the peaks which intensities are localized in connected regions smallest than `minNumPixels` within the ROI. Different aggressiveness levels determine whether also the connected components outside the ROI should be evaluated. Pixel size is equal to about 100um. Here, we are interested in all the sub-regions with an area larger than 90000um^2 Different levels of aggressiveness can produce different results.

We will set `aggressive` equal to 1.

```
## Count filter selects the peaks with a signal localized in clusters of pixels
## of size larger than "minNumPixels". The signal pixels are extracted applying
## Otsu's thresholding to the peak intensity image. Here, we select peaks that
## are connected in regions larger than 9 pixels. This parameter depends on the
## size of expected realistic sub-regions, and it is closely connected with the
## physical pixel size.

start.time <- Sys.time()
res.count.filter <- countPixelsFilter(msiData = msiX,
                                      roiImage = ref.roi$ROI,
                                      minNumPixels = 9,
                                      aggressive = 1,
                                      verbose = FALSE)
Sys.time() - start.time
```

```
## Time difference of 8.169137 secs
```

```r
print(length(res.count.filter$sel.peaks))
```

```
## [1] 601
```

```r
## Apply the filter - the second element of the previosly calculated list
## corresponds to aggressive = 1
msiX <- applyPeaksFilter(msiX, res.count.filter)
```

Complete spatial randomness filter

A filter based on testing for complete spatial randomness (CSR) is applied on the spatial distribution of the binarized peaks signals. Here we apply the Clark Evans test `"ClarkEvans"` to check whether the pixel distribution follows the density of the covariate (calculated, in this case, as the smoothed TIC image). The Bonferroni-corrected p-values are thresholded to 0.001 in order to select the non-randomly scattered peaks.

```r
## Here, we select the peaks for which the corresponding point pattern process
## (see spatstat::ppp for more details) hypothesis of complete spatial randomness
## can be rejected. In this case, we use the Clark Evans test, additional
## arguments, compatible with spatstat::clarkevans.test can be passed to the
## function. The p-values are corrected using Bonferroni correction.
res.csr.stats <- CSRPeaksFilter(msiData = msiX,
                                method = "ClarkEvans",
                                calculateCovariate = FALSE,
                                adjMethod = "bonferroni",
                                returnQvalues = TRUE,
                                plotCovariate = FALSE,
                                verbose = TRUE)
```

```
## Calculating the reference image. This may take a while...
## Scaling all the peaks intensities in [0, 1]
## Starting CSR tests. This may take a while...
## 500
```

```r
## Set a threshold of 0.001 for rejection of complete spatial randomness.
cat(sum(res.csr.stats$p.value < 0.001), "\n")
```

```
## 454
```

```r
cat(sum(res.csr.stats$q.value < 0.001), "\n")
```

```
## 398
```

Apply the filtering

```r
## Select the peaks for which the null hypothesis of complete spatial randomness
## can be rejected, using the Bonferroni corrected q-values.
csr.filt <- createPeaksFilter(which(res.csr.stats$q.value < 0.001))

msiX <- applyPeaksFilter(msiX, csr.filt)
```

Comparison between the original and filtered datasets

Here we extract the first 3 principal components and visualize them as RGB channels. The scores are first scaled in [0, 1].

```r
## PCA original dataset
start.time <- Sys.time()
pca.orig <- prcomp(desiData)
Sys.time() - start.time
```

```
## Time difference of 45.26087 secs
```

```r
## PCA filtered dataset
start.time <- Sys.time()
pca.filt <- prcomp(getIntensityMat(msiX))
Sys.time() - start.time
```

```
## Time difference of 3.708076 secs
```

```r
## Plot the first 3 principal components as RGB channels
im.orig.rgb <- array(NA, c(sz[1], sz[2], 1, 3))
for (i in 1:3) {
  im.orig.rgb[, , 1, i] <- matrix((pca.orig$x[, i] - min(pca.orig$x[, i])) /
                                    (max(pca.orig$x[, i]) - min(pca.orig$x[, i])),
                                  sz[1], sz[2])[, sz[2]:1]
}
im.filt.rgb <- array(NA, c(sz[1], sz[2], 1, 3))
for (i in 1:3) {
  im.filt.rgb[, , 1, i] <- matrix((pca.filt$x[, i] - min(pca.filt$x[, i])) /
                                    (max(pca.filt$x[, i]) - min(pca.filt$x[, i])),
                                  sz[1], sz[2])[, sz[2]:1]
}
par(mfrow = c(1, 2), mar = c(1, 0.2, 1, 0.2))
plot(as.cimg(im.orig.rgb), main = "First 3 PC, original data")
plot(as.cimg(im.filt.rgb), main = "First 3 PC, filtered data")
```

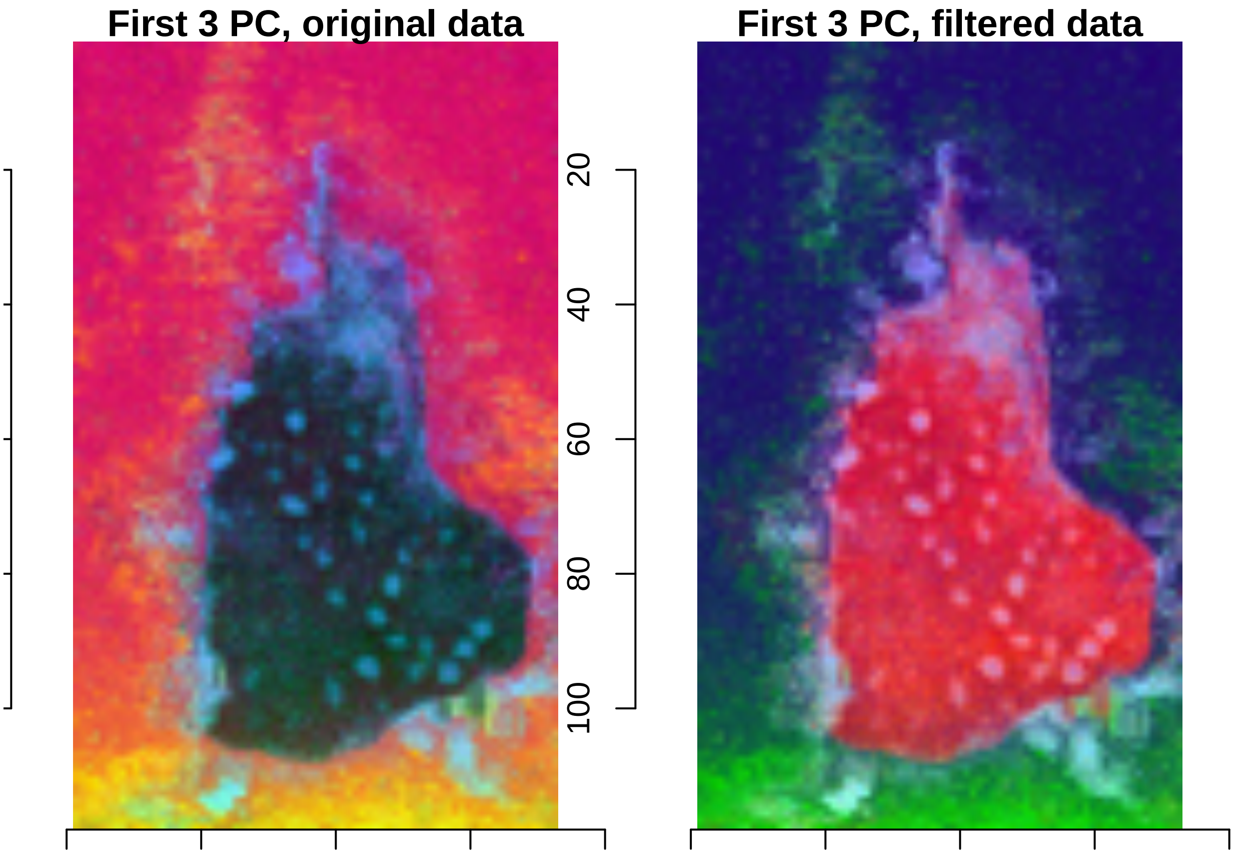

Here we compare the clustering results using k-means with a number of clusters equal to 5 on the first K principal components scores responsible of the 95% of the total variance.

```
## Determine the number of principal components responsible of the 95% of the
## total variance
var.perc.orig <- cumsum(pca.orig$sdev^2 / sum(pca.orig$sdev^2))
var.perc.filt <- cumsum(pca.filt$sdev^2 / sum(pca.filt$sdev^2))
num.comps.orig <- which(var.perc.orig >= 0.95)[1]
num.comps.filt <- which(var.perc.filt >= 0.95)[1]
cat("Num. components original dataset =", num.comps.orig, "\n")
```

```
## Num. components original dataset = 358
```

```
cat("Num. components filtered dataset =", num.comps.filt, "\n")
```

```
## Num. components filtered dataset = 187
```

```
start.time <- Sys.time()
clust.orig <- kmeans(pca.orig$x[, 1:num.comps.orig], centers = 5,
                     iter.max = 1000, nstart = 50)
Sys.time() - start.time
```

```
## Time difference of 18.15142 secs
```

```
start.time <- Sys.time()
clust.filt <- kmeans(pca.filt$x[, 1:num.comps.filt], centers = 5,
                     iter.max = 1000, nstart = 50)
```

```
## Warning: Quick-TRANSfer stage steps exceeded maximum (= 430700)
```

```
## Warning: Quick-TRANSfer stage steps exceeded maximum (= 430700)
```

```
Sys.time() - start.time
```

```
## Time difference of 14.29672 secs
```

```
## Visualize the clusters
par(mfrow = c(1, 2), mar = c(1, 0.2, 1, 0.2))
image(matrix(clust.orig$cluster, sz[1], sz[2]), col = viridis::inferno(5),
      main = "K-means orig. dataset")
image(matrix(clust.filt$cluster, sz[1], sz[2]), col = viridis::inferno(5),
      main = "K-means filt. dataset")
```

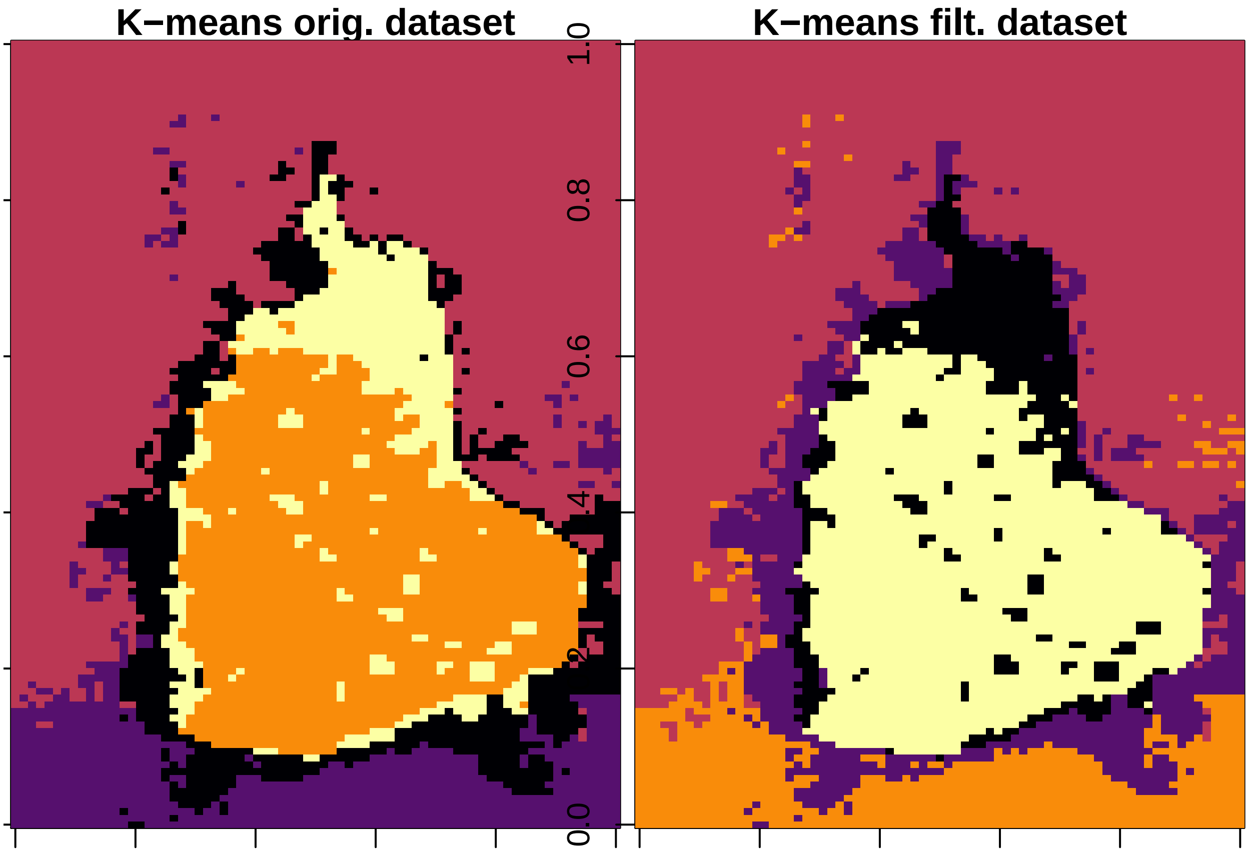

Finally, let's check the image corresponding to the smallest number of signal pixels.

```
## Calculate the fraction of signal pixels for each peak.
num.pixels <- nrow(getIntensityMat(msiX))
frac <- array(0, ncol(getIntensityMat(msiX)))
for (i in 1:length(frac))
{
  frac[i] <- sum(getIntensityMat(msiX)[, i] != 0) / num.pixels
}

## The smallest sub-region
print(min(frac))
```

```
## [1] 0.005688414
```

```r
## Visualize the peak image corresponding to the smallest sub-region
im.smallest <- matrix(getIntensityMat(msiX)[, which.min(frac)],
                      getShapeMSI(msiX)[1], getShapeMSI(msiX)[2])

plot(msImage(im.smallest,
             name = as.character(round(getMZ(msiX)[which.min(frac)], 4))
             )
     )
```

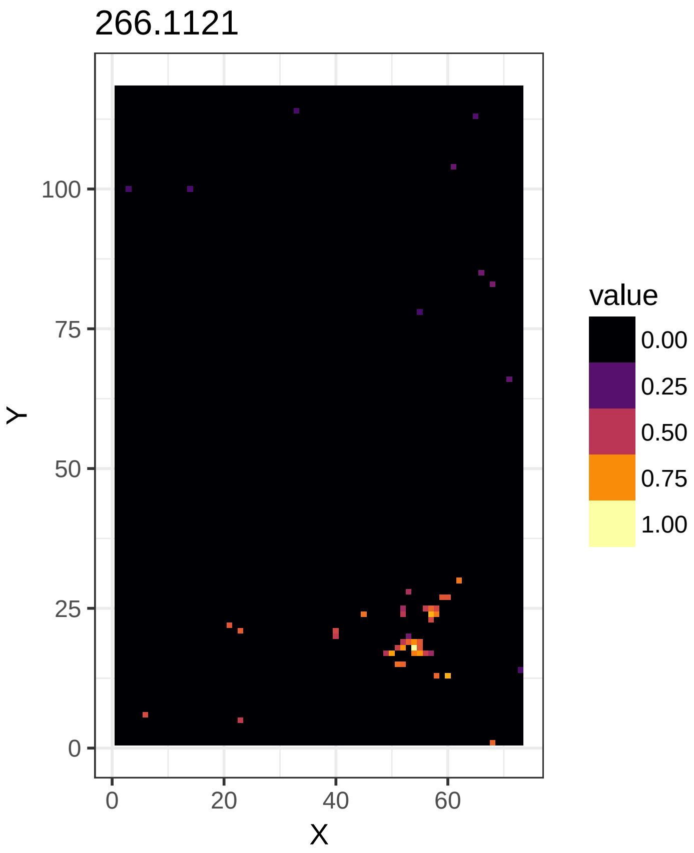

Clearly, this intensity image shows a non-random pattern that would have been removed by a naive fraction filter with a threshold of 1%.

References

Gibb, Sebastian, and Korbinian Strimmer. 2012. "MALDIquant: A Versatile R Package for the Analysis of Mass Spectrometry Data." *Bioinformatics* 28 (17). Oxford University Press: 2270–1.
